# Association of residual feed intake with intestinal microbiome and metabolome in laying period of ducks

**DOI:** 10.3389/fmicb.2023.1138914

**Published:** 2023-05-12

**Authors:** Hanxue Sun, Wenwu Xu, Tiantian Gu, Jing Sun, Chengfeng Li, Li Chen, Yong Tian, Guoqin Li, Lizhi Lu, Tao Zeng

**Affiliations:** ^1^State Key Laboratory for Managing Biotic and Chemical Threats to the Quality and Safety of Agro-products; Key Laboratory of Livestock and Poultry Resources (Poultry) Evaluation and Utilization, Ministry of Agriculture and Rural Affairs, Institute of Animal Husbandry and Veterinary Medicine, Zhejiang Academy of Agricultural Sciences, Hangzhou, China; ^2^Institute of Animal Husbandry and Veterinary Medicine, Hubei Academy of Agricultural Sciences, Wuhan, China; ^3^Hubei Shendan Health Food Co., Ltd., Xiaogan, China

**Keywords:** residual feed intake, laying duck, production performance, microbiome, metabolome

## Abstract

**Introduction:**

Residual feed intake (RFI) is a indicator to evaluate animal feed. This experiment was explored to study the relationship between intestinal microbiome and metabolome of ducks with different residual feed intake during laying period.

**Methods:**

A total of 300 Shaoxing ducks aged 42 weeks were randomly selected and fed a diet of 60 d. At the end of the trial, 20 samples were selected according to the phenotype of RFI and divided into two groups (HRFI and LRFI). The cecal microbiota composition was explored by 16S ribosomal RNA gene sequencing and rectal metabolomics uses liquid chromatography-mass spectrometry (LC–MS) to identify the composition of metabolites in a non-targeted manner.

**Results:**

Results show feed intake and feed conversion ratio in the group HRFI were significantly higher than those in the group LRFI (*p* < 0.05). Chao1 indices were higher in the group LRFI than in the HRFI (*p* < 0.05), Shannon and Simpson indices were higher in the group LRFI than in the HRFI (*p* < 0.01). After linear discriminant analysis effect size (*p* < 0.05, LDA score > 3), *Rikenellaceae*, *Rikenellaceae_RC9_gut_group*, *Lactobacillales* and *Ruminococcus_2*, etc. were significantly enriched in the group LRFI at the genus level, while *Prevotellaceae_NK3B31_group* and *Bacteria* were significantly enriched in the group HRFI. After LC–MS analysis we found 338 metabolic difference products and 10 metabolic pathways, including the ABC transporter system, cysteine and methionine metabolism, arginine and proline metabolism, and vitamin B6 metabolism, were identified to be associated with the significantly differentially expressed between the groups LRFI and HRFI (*p* < 0.05). We hypothesize that the difference between ducks with different RFIs is mainly due to the fact that ducks with LRFI have more SCFAs-producing bacteria in their gut microorganisms, which regulate the RFI of animals. This process we found that *Phascolarctobaterium* and *Anaerobiospirillum* may provide energy for ABC transporter system by producing SCFAs, and regulate RFI to improve feed utilization efficiency.

**Discussion:**

These results revealed the relationship between microbiome and metabonomics in laying ducks with different RFI, and provided theoretical basis for further study on the relationship between them.

## Introduction

Feed is the largest cost of production in the duck industry in China, and meeting feed requirements is very important for successful duck breeding (Zeng et al., 2016). The main components of the diet of laying ducks are corn, wheat, and soybean; due to the scarcity of biofuels, some countries are using farmland for biofuel production (Hill et al., 2006; Godfray et al., 2010), and climate change and other factors are driving up costs (Tigchelaar et al., 2018). The costs of duck production will consistently increase because of the reliance on the feed ingredients, and natural factors. Therefore, improving the efficiency of converting feed of ducks can greatly ameliorate this existing circumstances. Residual food intake (RFI) is the difference between the food intake of animals in actual production and the expected food intake calculated by people at this growth stage, which is used to evaluate the efficiency of animals on feed (Aggrey et al., 2010; Wen et al., 2018).

The intestinal microorganism is considered as an organ of the body and plays an important role in host feed digestion, nutrient absorption and energy supply (McCormack et al., 2017). Many studies have shown an important link between feed utilization efficiency and host gut microflora. Xu et al. (2021) found that the addition of 1 × 10^9^ CFU/kg *Clostridium butyricum* to the diet significantly increased the relative abundance of *Clostridium butyricum* and *Lactobacillus* in the broiler intestine, up-regulated the expression levels of genes related to intestinal barrier function, and improved feed utilization efficiency. Wen et al. (2021) found that the contribution of flora to the RFI phenotype was different in different intestinal parts of chickens, with cecum microorganisms contributing up to 28%. It has also been shown that this difference is due to different genetic selection for digestive efficiency, but still the role of microbial regulation of RFI cannot be denied (Borey et al., 2020). It has been proved that microorganisms can degrade polysaccharide and fiber in feed and improve feed efficiency of chickens (Zhang et al., 2022). Li et al. (2021) study show that feed restriction could change the microbial community of duck intestines, it further indicates that the feed intake will affect the intestinal flora of animals. The gut microbiota has been shown to be closely related to feed efficiency in poultry (Stanley et al., 2016; Metzler-Zebeli et al., 2019). Metabolic processes play an important role in animals, involved in the breakdown or synthesis of many important molecules (Wang and Kadarmideen, 2019). Furthermore, compared to DNA, RNA, and proteins, metabolites are closer to observable phenotypes. Therefore, metabolites are highly correlated with RFI and can be used in the selection of low RFI animals (feed-efficient animals) for better saving cost of feed or breeding (Wang and Kadarmideen, 2019).

However, the relationship between the gut microbiome and metabolome of laying ducks with different residual feed intake, and the relationship between the two and poultry feed efficiency remains unclear (Mignon-Grasteau et al., 2015). Through comprehensive analysis of the intestinal microbiome and metabolome, the relationship between them and RFI can be better explained. Therefore, in this study, we assessed the effects of different RFIs on production performance, gut microbiota and metabolome of Shaoxing ducks, and investigated the association between them.

## Materials and methods

### Experimental design

A total of 300 Shaoxing ducks aged 42 weeks were randomly selected fed a diet for 60 d. Feed intake (FI) was the weight of feed taken by animals within 24 h. Egg mass laid (EML), body weight (BW, average weight before and after the trial), and ∆W (BW gain) were measured. We estimate that the MMW and RFI of ducks are relationship, and the specific methods and parameters are described in our previous articles (Zeng et al., 2016). At the end of the experimental period, we selected 10 extreme individuals (20 in total) according to different residual feed intake, and divided them into two groups to represent two different RFI performances (High RFI, HRFI and low RFI, LRFI). There was only significant difference in residual feed intake between the two groups, but no difference in other phenotypes.

### Experimental materials and feeding management

The experiment was carried out at the national breeding site of Shaoxing Duck in Zhejiang Province, China. The ducks were kept in a three-story cage, and each cage position is separated by a partition to avoid experimental errors caused by pecking at each other’s feed. The basic feed of the experimental ducks combined with the needs of ducks during the egg-laying period met the nutritional requirements of the Chinese egg and duck standard (GB/T 41189–2021), and the basic composition of the feed was shown in Supplementary Table S1.

### Sample collection

The daily individual FI, egg laying weight, initial weight, and end weight were recorded, and the RFI was calculated. The highest ranked 10 and the lowest ranked 10 were assigned to the groups HRFI and LRFI, respectively. Measured the egg shape index (Vernier caliper, Deguqmnt), eggshell thickness (ETG-1061 eggshell thickness gauge, Robotmation), shell strength (EFG-0503 eggshell strength gauge, Robotmation, Tokyo, Japan), and egg weight, yolk color, albumen height, Haugh unit were used to define internal egg quality (EMT-5200 egg quality gauge, Robotmation) of the two groups of eggs, respectively. After euthanasia of each group of ducks, the contents of the cecum and rectum were collected and stored at −80°C for analysis.

### Cecum microbiome

Extraction of DNA from Intestinal Contents Using the DNA Template Mini Kit (Qiagen, Hilden, Germany). The 16S rRNA gene was amplified by PCR with primers, and the detected region was V3-V4 region 341F: (5’-CCTACGGGNGGCWGCAG-3′) and the reverse primer 805R: (5’-GACTACHVGGGTATCTAATCC-3′). The construction of the gene library is based on Ion Plus Fragment Library Kit (48 rxns) (Thermo Fisher Scientific, United States). A single-ended sequencing platform is then used which performed using the IonS5 platform at Zhejiang Tianke Hi tech Development Co., Ltd. (Hangzhou, China).

### Sequencing data analysis

The microbiome bio-information analysis section was performed using QIIME2 2019.4. The reads sequence is compared with the Gold database^1^ and UCHIME Algorithm^2^ to remove the chimeric sequence to obtain the clean data. Clustering was performed using Uparse (version 8.1.1861, http://drive5.com/uparse/) (Edgar et al., 2011) with 97% similarity, and representative operational taxonomic units (OTUs) sequences were selected (Wang et al., 2007). The phylogenetic affiliation of each 16S rRNA gene sequence was analyzed by the ribosome database project classifier^3^ against the Silva (version 138.1, http://www.arb-silva.de) 16S rRNA database using confidence threshold of 80% (DeSantis et al., 2006).

The rarefaction analysis based on Mothur (version 1.30.1) (Schloss et al., 2009) was conducted to reveal the diversity indices, including the Chao1, Shannon index, Simpson index, and Goods coverage diversity indices. The beta diversity analysis was performed using UniFrac (Oksanen et al., 2015) to compare the results of the principal component analysis (PCA). Linear discriminant analysis effect size (LEfSe) analysis was used to identify the different bacterial populations of the two groups (Ijaz et al., 2018).

### Metabolomics profiling for rectal contents

Pre-frozen specimens were added 100 mg glass bead, placed in a tissue grinder mechanically ground, then added a mixture of CH2Cl2/MeOH (1:1, v/v) (stored at −20°C). After centrifugal filtration transfer into the detection bottle for liquid chromatography–mass spectroscopy (LC–MS) detection. The LC analysis was four times using hydrophilic-interaction chromatography (HILIC) and reverse phase liquid chromatography (RPLC) separation in both positive and negative ionization modes. Based on the non targeted metabonomics of Nexera UPLC Ultra High Performance Liquid Phase Tandem QE High Resolution Mass Spectrometer, combined with the metabonomics data processing software Progenisis QI v2.3, the qualitative and relative quantitative analysis of the original data was carried out, and the original data were standardized and preprocessed (Sun et al., 2021).

### Statistical analysis

Feed efficiency traits and egg quality traits were analyzed in SPSS software package (SPSS version 22.0; IBM Corp, Armonk, NY, United States). Shapiro–Wilk test was used for phenotypic aspects of feed efficiency data, and Student’s t-test was used to analyze differences after compound normal distribution conditions. The Kruskal-Wallis rank sum test was used to select and demonstrate differentially abundant taxa between the groups LRFI and HRFI. Correlations between different cecal microbial genera and differentially expressed metabolites in the rectal samples were assessed using Spearman’s correlation test. *p* < 0.05 was considered significant.

## Results

### Differences in phenotypes of laying ducks with different RFI

Difference analysis showed (Table 1) that FI and FCR were significantly higher in the group HRFI than in the group LRFI (*p* < 0.05). There were no differences in EML, BW, and ∆W between the groups HRFI and LRFI (*p* > 0.05). There were no differences in the egg quality traits between the LRFI and HRFI ducks (*p* > 0.05).

**Table 1:** Means (±SE) of feed efficiency and egg quality traits in a duck population divergently selected for low (LRFI) or high (HRFI) residual feed intake.

Traits^a^	HRFI groups (*n* = 10)	LRFI groups (*n* = 10)	*p*-value^b^
Feed efficiency traits			
RFI (g/day)	20.1 ± 3.6	−25.8 ± 2.3	/
FI (g/day)	184.6 ± 0.8	141.8 ± 2.9	<0.01
EML (g/day)	66.4 ± 1.3	66.0 ± 2.1	0.63
BW (g)	1354.8 ± 38.5	1338.3 ± 44.7	0.42
ΔW (g/day)	1.7 ± 0.8	1.4 ± 1.0	0.37
FCR	2.8 ± 0.1	2.2 ± 0.1	<0.01
Egg quality traits			
Daily egg mass (g)	75.38 ± 1.76	73.12 ± 1.49	0.73
Egg shape index (%)	1.34 ± 0.01	1.36 ± 0.02	0.21
Shell thickness (mm)	0.51 ± 0.02	0.52 ± 0.01	0.53
Shell strength (kg/cm^2^)	4.93 ± 0.17	5.19 ± 0.11	0.19
Yolk color	11.71 ± 0.09	11.88 ± 0.11	0.37
Albumen height (mm)	6.82 ± 0.31	6.42 ± 0.17	0.20
Haugh unit	76.92 ± 2.15	75.13 ± 1.17	0.48

a FI, feed intake; EML, egg mass laid; BW, body weight; ΔW, body weight gain; FCR, feed conversion ratio; RFI, residual feed intake.

b *p*-value obtained by t-test.

### Bacterial analyses of cecal contents

**Figure 1:**
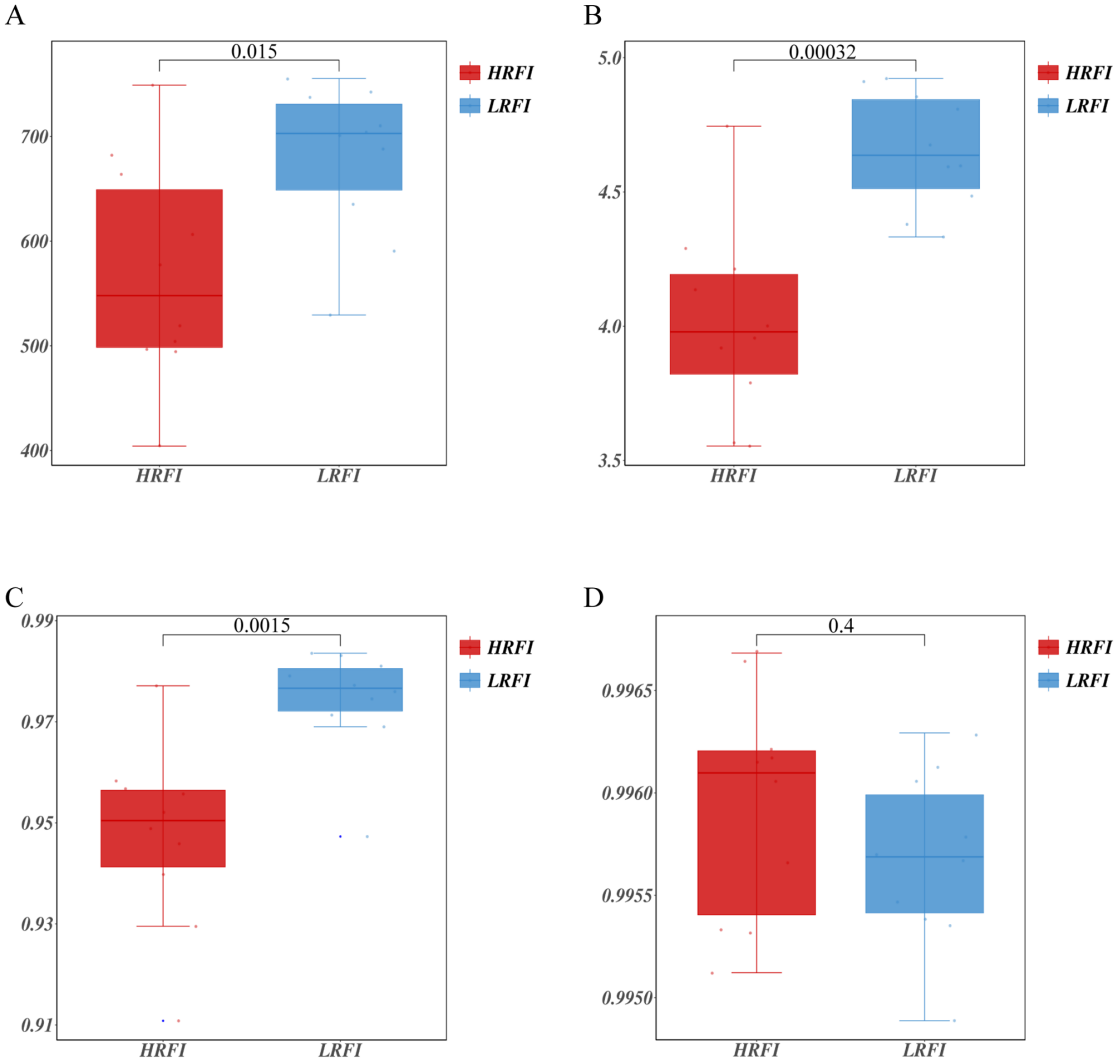
Measurements of alpha diversity in the cecal microbiota at the OTU level using Chao1, Shannon index, Simpson index, and Goods coverage indices in the groups HRFI and LRFI. Chao1 **(A)**; Shannon index **(B)**; Simpson index **(C)**; Goods coverage **(D)**.

A total of 1,064,989 bases (Supplementary Table S2) were obtained from 20 samples. After quality inspection and removal of chimeric sequences, the average number of readings produced from each duck’s cecal sample was 41,162 (Supplementary Table S2). A total of 1,017 OTUs were obtained and successfully classified to the domain level using classifiers. Rarefaction analyses were performed to gauge for adequate sequencing depth per sample (Supplementary Figure S1). The α diversity assessed using Chao1, Shannon index, Simpson index, and Goods coverage indices is shown in Figure 1 (Supplementary Table S3). Chao1 indices were higher in the LRFI than in the group HRFI (*p* < 0.05) (Figure 1A), Shannon, and Simpson indices were higher in the LRFI than in the group HRFI (*p* < 0.01) (Figures 1B,C), suggesting that cecal microflora richness and diversity was higher in the group LRFI than in the HRFI.

**Figure 2:**
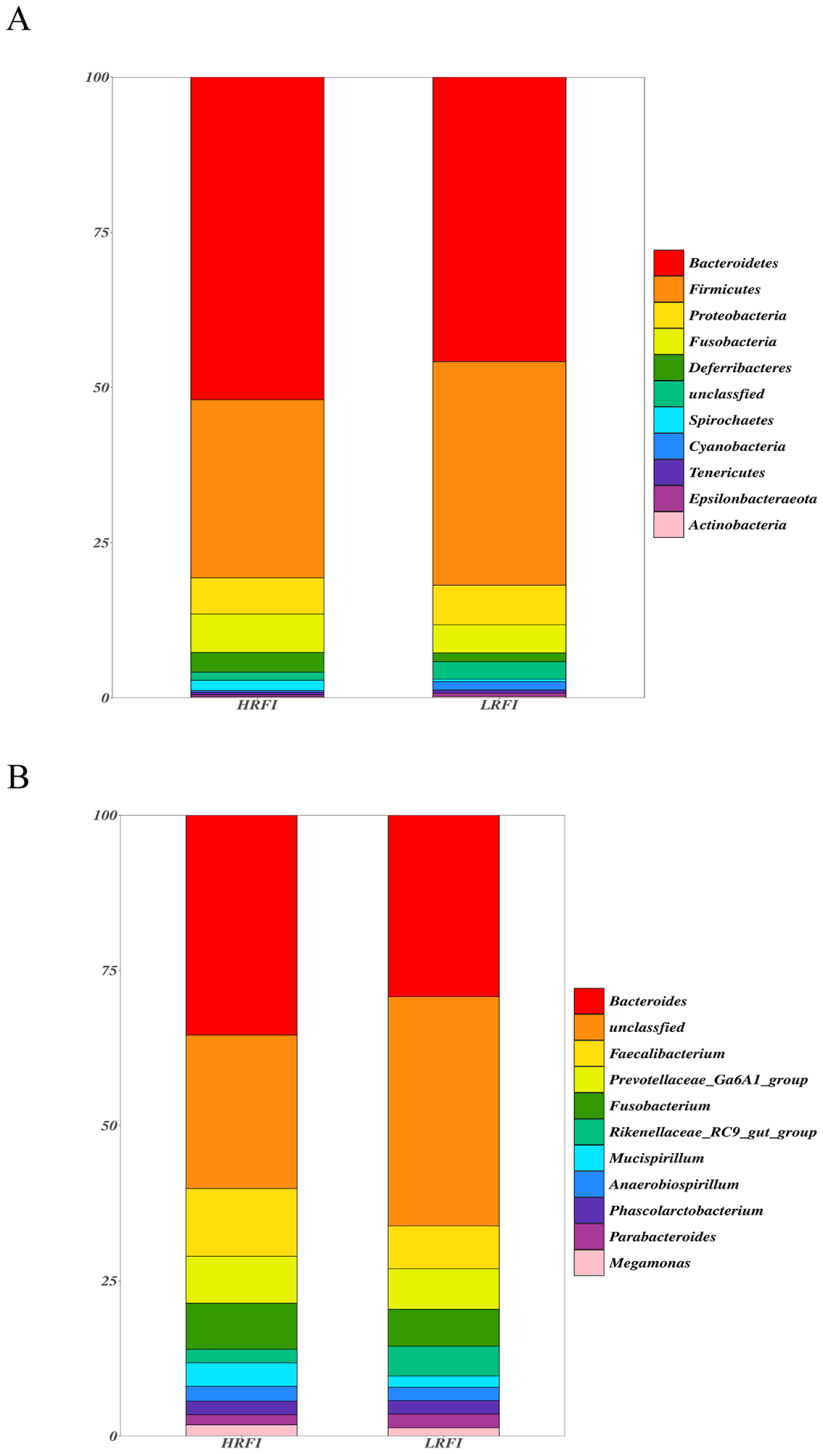
Taxonomic classification of the gut microbial composition in each group at the phylum and genus level: phylum level **(A)**; genus level **(B)**.

We identified top 10 bacterial phyla and genus in the cecal microflora of the duck (Figure 2). The community structure of cecal microflora in samples from the groups LRFI and HRFI was nearly identical at the phylum level. At the phylum level (Figure 2A), in the group HRFI, *Bacteroidetes* were the most abundant bacteria (52.21%), followed by *Firmicutes* (28.83%), *Proteobacteria* (5.82%), and *Fusobacteria* (6.22%). In the group LRFI, *Bacteroidetes* were the most abundant bacteria (48.49%), followed by *Firmicutes* (37.07%), *Proteobacteria* (6.41%), and *Fusobacteria* (2.25%). The abundances of *Firmicutes* and *Proteobacteria* in the group LRFI were higher than in the HRFI, and the abundances of *Bacteroidetes* and *Fusobacteria* in the group LRFI were lower than in the HRFI. At the genus level (Figure 2B), in the group HRFI, *Bacteroides* were the most abundant bacteria (35.95%), followed by *Faecalibacterium* (11.07%), *Prevotellaceae_Ga6A1_group* (7.68%), and *Fusobacterium* (7.52%). In the group LRFI, *Bacteroides* were the most abundant bacteria (26.92%), followed by *Faecalibacterium* (6.35%), *Prevotellaceae_Ga6A1_group* (5.99%), and *Fusobacterium* (5.47%). The abundances of *Megamonas* in the group LRFI were higher than in the HRFI, and the abundances of *Anaerobiospirillum* in the group LRFI were lower than in the HRFI.

**Figure 3:**
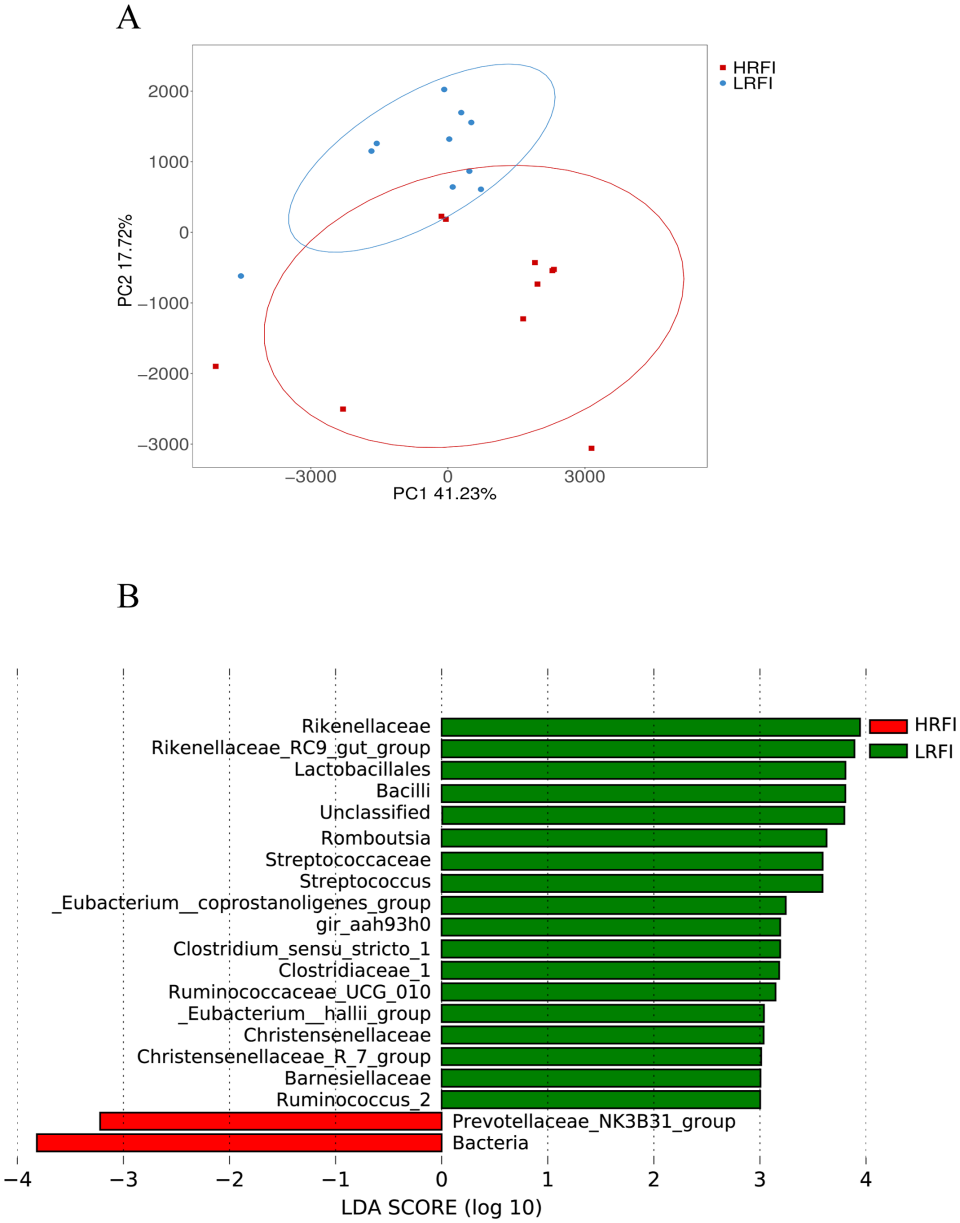
Principal coordinate analysis (PCA) of taxonomical classifications of cecal bacterial communities of ducks **(A)**; LEfSe analysis with LDA threshold 3 **(B)**.

PCA analysis revealed a clear structural difference in the composition of gut microbiota between high and low RFI ducks (Figure 3A). Differentially abundant taxa between HRFI and LRFI were also identified using LEfSe analysis (*p* < 0.05, LDA score > 3). *Rikenellaceae, Rikenellaceae_RC9_gut_group, Lactobacillales, Bacilli, Romboutsia, Streptococcaceae, Streptococcus, Eubacterium__coprostanoligenes_group, gir_aah93h0, Clostridium_sensu_stricto_1, Clostridiaceae_1, Ruminococcaceae_UCG_010, Eubacterium__hallii_group, Christensenellaceae, Christensenellaceae_R_7_group, Barnesiellaceae, and Ruminococcus_2* were enriched in LRFI (*p* < 0.05), *Prevotellaceae_NK3B31_group* and *Bacteria* were enriched in HRFI (*p* < 0.05) (Figure 3B).

### Metabolomic analyses of rectal contents

**Figure 4:**
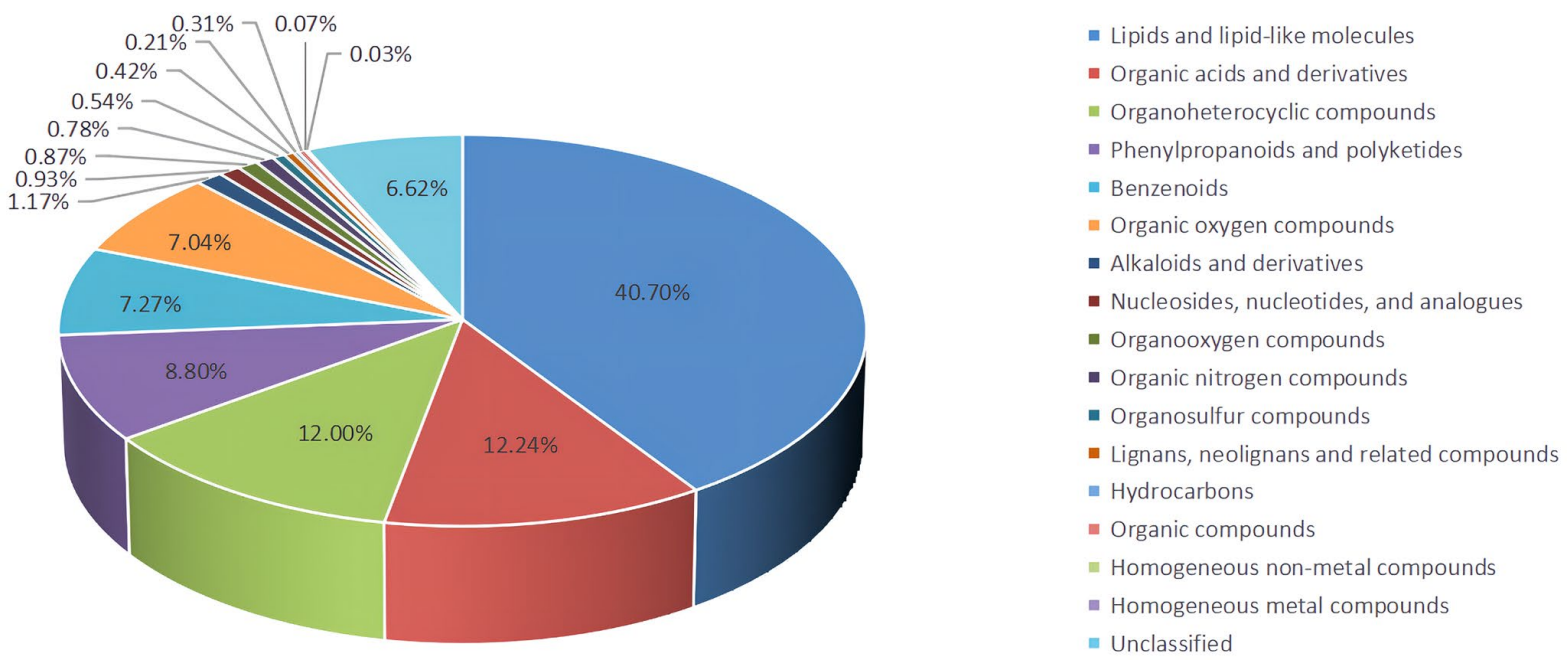
The proportion of identified metabolites in metabolome analysis.

Untargeted LC–MS identified 6,674 metabolites, including 40.70% lipids and lipid-like molecules, 12.24% organic acids and derivatives, and 12.00%, 8.80%, 7.27%, and 7.04 organoheterocyclic compounds, phenylpropanoids and polyketides, benzenoids, organic oxygen compounds and alkaloids and derivatives, respectively, and 6.62% undefined metabolites (Figure 4).

**Figure 5:**
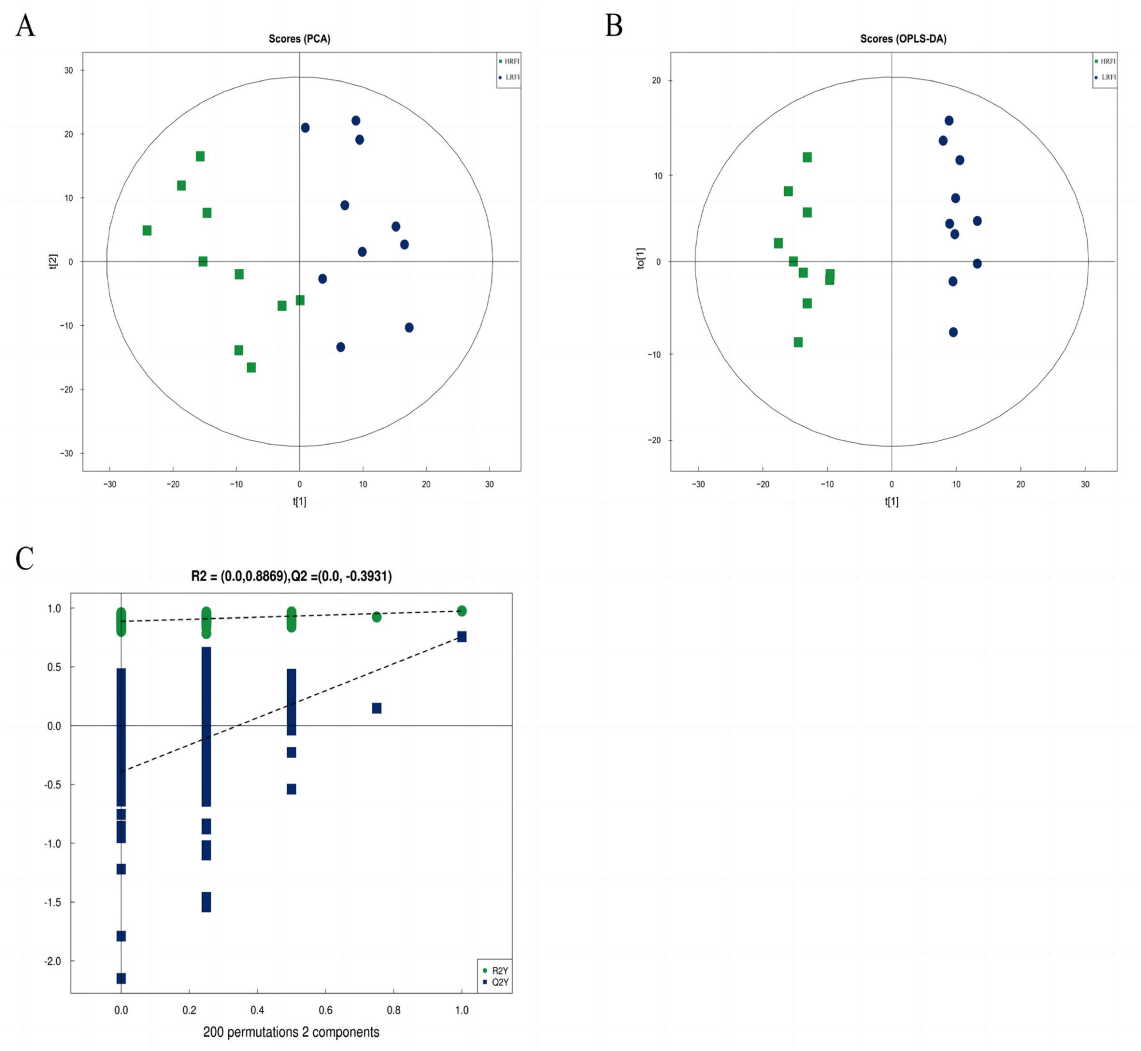
The score scatter plot of PCA model for the groups LRFI vs. HRFI **(A)**. The score scatter plot of the OPLS-DA model for the groups LRFI vs. HRFI **(B)**. The permutation test of the OPLS-DA model for the groups LRFI vs. HRFI **(C)**.

The score scatter plot of PCA and OPLS-DA models of rectal metabolism in laying ducks with different residual feed intake is shown in Figures 5A,B and Supplementary Table S4. R^2^X for PCA was greater than 0.5, showing noticeable separations between the groups LRFI and HRFI. Besides that, the Q^2^ on the Y-axis was negative (0.3947) in the random-permutation test (Figure 5C; Supplementary Table S4), indicating that the models did not overfit.

**Figure 6:**
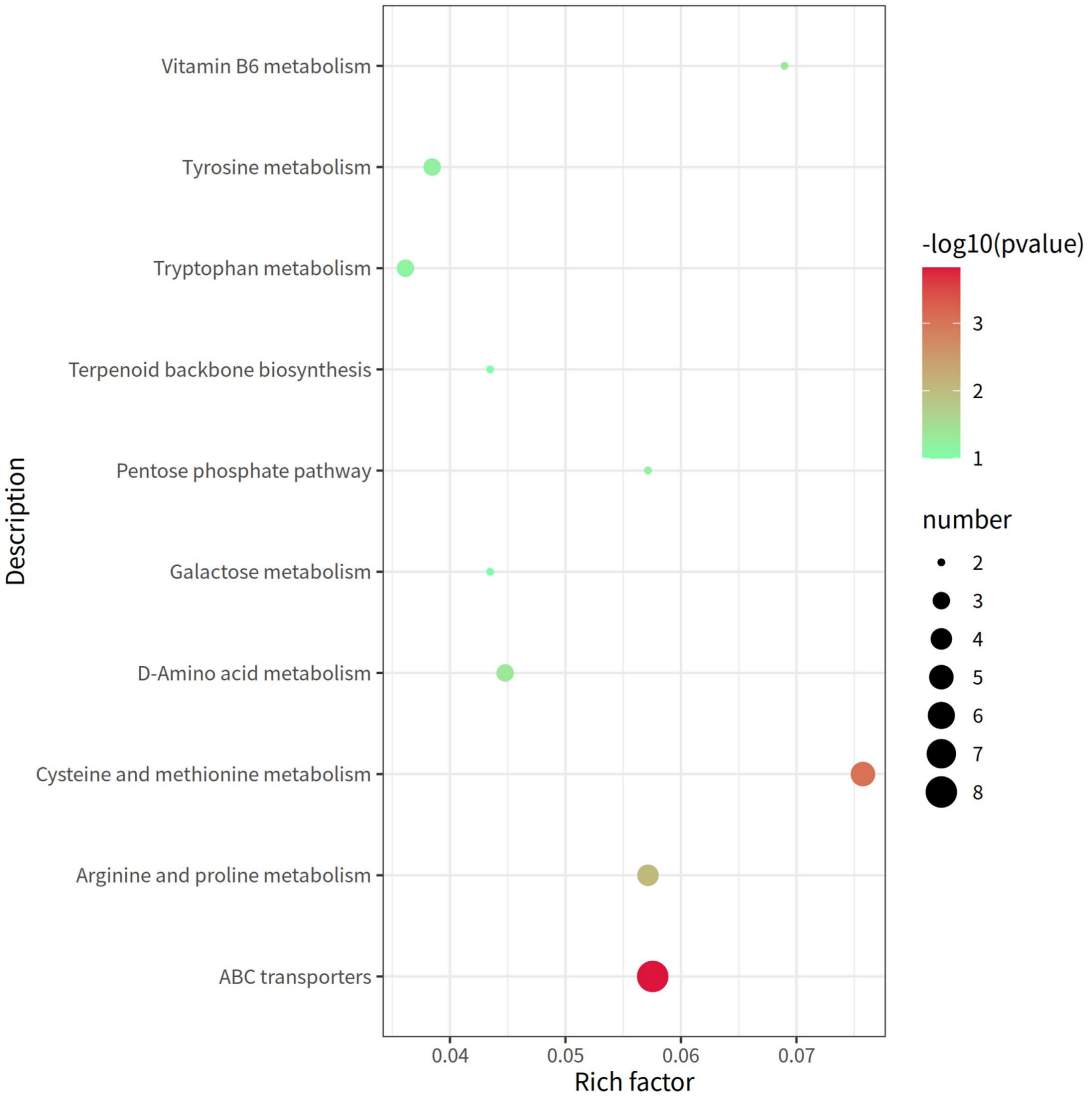
The rectal metabolomics pathway of two groups of laying ducks with different residual feed intake was analyzed. The bubbles in the figure represent the effect of different residual feed intake on the metabolic pathway of the sample; the larger the bubbles, the redder the color, the greater the effect on the pathway.

A total of 338 metabolites (158 in the negative mode and 180 in the positive mode) in rectal contents were remarkable differentially expressed between the groups LRFI and HRFI (Supplementary Table S5). Ten metabolic pathways, including the ABC transporter system, cysteine and methionine metabolism, arginine and proline metabolism, and vitamin B6 metabolism, were identified to be associated with the significantly differentially expressed metabolites between the LRFI and HRFI groups (Figure 6).

### Correlation between the cecal microbiome and rectal metabolome

**Figure 7:**
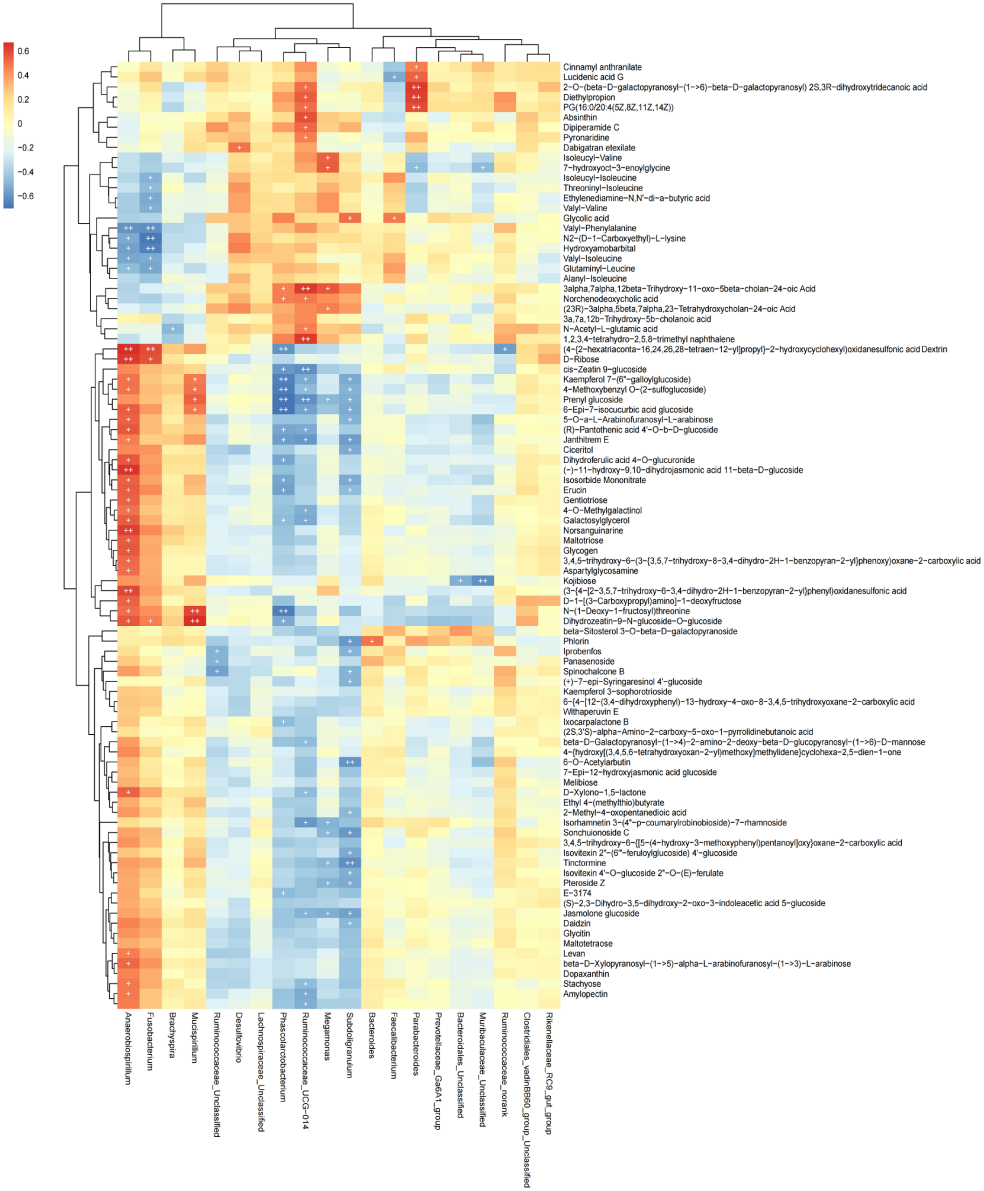
Heat map showing the correlation between microbial species and metabolites. Positive correlation is shown in red; negative correlation is shown in blue. The darker the color, the stronger the correlation, + is used to indicate that “species-metabolite” and significant correlation (*p* < 0.05).

The functional correlations between different microbial genera and the significantly differentially expressed metabolites in rectal samples were determined using Spearman’s correlation analysis (Figure 7). We found 93 differentially expressed metabolites to be correlated with 20 genera. The abundance of *Phascolarctobaterium* was significantly negatively correlated with Kaempferol 7-(6″-galloylglucoside), 4-Methoxybenzyl O-(2-sulfoglucoside), Prenyl glucoside, 5-Epi-7-isocucurbic acid glucoside and N-(1-Deoxy-1-fructosyl) threonine (*p* < 0.05). The abundance of *Anaerobiospirillum* was significantly positively correlated with Norsanguinarine, (3-[4-[2–3.5,7-trihydroxy-6-3,4-dihydro-2H-1-benzopyran-2-yllphenyl)oxidanesulfo-nic acid, (−)-11-hydroxy-9,10-dihydrojasmonic acid 11-beta-D-glucoside and (4-{2-hexatriaconta-16,24,26,28-tetraen-12-yl]propyl}-2-hydroxycyclohexyl)oxidanesulfonic acid Dextrin D-Ribose (*p* < 0.05). These data indicated that cecal microbial composition affected metabolite expression.

## Discussion

In recent years, with the improvement of breeding environment and the acceleration of breeding process, feed efficiency has been greatly improved (Bezerra et al., 2013; Zeng et al., 2018). This experiment refers to previous research methods and groups according to different RFI (Baker et al., 2006; Nkrumah et al., 2007). We found that the FI and FCR of egg duck in the group LRFI were significantly lower than that of egg duck in the group HRFI, indicating that LRFI feed wastes were less and feed conversion rates were higher. This is consistent with previous research (Yuan et al., 2015; Zeng et al., 2016). The shell thickness, shell strength, yolk color and Haugh unit are important indicators of egg quality (Ahammed et al., 2014). In our experiment, there were no differences in the egg quality traits between the LRFI and HRFI ducks.

The cecal microbiota composition significantly impacts the growth and health of poultry (Lunedo et al., 2019; Wang et al., 2021). The intestinal microflora of poultry mainly exists in the cecum, so the cecum plays an key role in the digestion and absorption of poultry (Liu et al., 2016). We found that Chao1, Shannon index, Simpson indices were higher in the group LRFI than in the HRFI. Therefore, cecal microflora richness and diversity were higher in the group LRFI.

*Bacteroidetes* and *Firmicutes* were predominant in the cecum microbiome of the groups LRFI and HRFI, consistent with previous studies (Yadav and Jha, 2019; Liu et al., 2021). The abundances of *Firmicutes* and *Proteobacteria* in the LRFI group were higher than in the HRFI, and the abundances of *Bacteroidetes* and *Fusobacteria* in the group LRFI were lower than in the HRFI. *Firmicutes* and *Bacteroidetes* have been shown to be the most important microbial phyla in the intestines of poultry (Lyu et al., 2021). In our study, FI and FCR were lower in the group LRFI than in the HRFI, but the abundance of *Firmicutes* were higher in the group LRFI than in the HRFI. It may be that *Firmicutes* plays a role in increasing the absorption of nutrients, and the FI and FCR of ducks in group LRFI are lower than those in HRFI. The metabolite of *Firmicutes* is mainly butyric acid, including members of the *Ruminococcus* and *Spirulina*, which can promote energy absorption in the gut (Chen and Xu, 2015; Komaroff, 2017; Louis and Flint, 2017), and metabolites of the genus *Bacteroides* can affect the absorption of nutrients by animals (Pittayanon et al., 2019; Robles-Vera et al., 2020). Butyric acid, a key substance for colon health and integrity, is the main metabolic substrate for coliforms, providing at least 60%–70% of their energy requirements for proliferation and differentiation (Sun et al., 2022). At the genus level, the abundances of *Megamonas* in the group LRFI were higher than in the HRFI and the abundances of *Anaerobiospirillum* in the group LRFI were lower than in the HRFI. A study speculate that *Megamonas* and *Anaerobiospirillum* may be involved in fat deposition and energy metabolism (Wang and Kadarmideen, 2019; Li et al., 2022). In our experiment, *Rikenellaceae* and *Ruminococcus_2*, etc. were significantly enriched in the group LRFI at the genus level, while *Prevotellaceae_NK3B31_group* and *Bacteria* were significantly enriched in the group HRFI. *Rikenellaceae* has been shown to have its metabolites involved in the degradation process of carbohydrates (Rowland et al., 2018), *Ruminococcus* has a similar function, regulating the degradation of polysaccharides and the production of short fatty acids (SCFAs) (Zhang et al., 2017), SCFAs stimulate the expression and secretion of appetite-affecting hormones in intestinal cells (Qin et al., 2021). Regulation of appetite and food intake induces satiety through neural and humoral pathways (Borgmann et al., 2021). It is hypothesized that in this experiment, gut microorganisms and their secondary metabolites (mainly SFACs) regulate the satiety of the animal organism, thus regulating the RFI of animals and improving feed utilization efficiency.

Metabolomics is the quantification of metabolites in the body, which can accurately reflect the changes in metabolic responses of organisms (Lao et al., 2009). In our study, the untargeted LC–MS approach assessed 6,674 metabolites in all samples and identified a total of 338 differentially expressed metabolites (158 in the negative mode and 180 in the positive mode) between the groups LRFI and HRFI. PCA and OPLS-DA results analysis confirmed that different RFI had a significant effect on the metabolism of ducks. The differentially expressed metabolites between the groups LRFI and HRFI were found to be associated with ten metabolic pathways, including the ABC transporter system, cysteine and methionine metabolism, arginine and proline metabolism, and vitamin B6 metabolism. A previous study showed that ABC transporters are effective in removing cholesterol from macrophages (Chen et al., 2021). Atherosclerosis intervention has also been shown to benefit from the upregulations of ABC transporters and Mer tyrosine kinase (Wang et al., 2022). Other researchers also suggest that inefficient animals are primarily associated with increased oxidative metabolism, which is possibly stimulated by increased oxidative stress (Alexandre et al., 2015; Tizioto et al., 2015). Vitamin B6 plays a key auxiliary role in multiple oxidative stress and metabolism (Johnstone et al., 2019; Ramos et al., 2019; Gholizadeh et al., 2020). In our study as the KEGG enrichment pathway map showed indicating that different RFI will affect the metabolic pathways of ABC transporters and Vitamin B6 metabolism in duck. Presumably because the intestinal SCFAs-producing bacteria supply energy to ABC transporters pathway, which in turn increases the intestinal absorption of nutrients to further improve feed utilization efficiency.

Through the integrated analysis of the microbiome and metabolome of Shaoxing ducks, we identified 93 differentially expressed metabolites to be correlated with 20 genera. We found 93 differentially expressed metabolites to be correlated with 20 genera. The abundance of *Phascolarctobaterium* was significantly negatively correlated with Kaempferol 7-(6″-galloylglucoside), 4-Methoxybenzyl O-(2-sulfoglucoside), Prenyl glucoside, 5-Epi-7-isocucurbic acid glucoside and N-(1-Deoxy-1-fructosyl) threonine. *Phascolarctobacterium* can produce SCFAs, including acetate and propionate, and may be related to the metabolic state of the host (Bajer et al., 2017). It also has anti-inflammatory roles (Trompette et al., 2014). Different RFI egg-type chickens have microbial communities in the cecum were significantly different (Yan et al., 2017). The abundance of *Anaerobiospirillum* was significantly positively correlated with Norsanguinarine, (3-[4-[2–3.5,7- trihydroxy-6-3,4-dihydro-2H-1-benzopyran-2-yllphenyl) oxidanesulfo-nic acid, (−)-11-hydroxy-9,10-dihydrojasmonic acid 11-beta-D-glucoside and (4-{2-hexatriaconta-16,24,26,28-tetraen-12-yl]propyl}-2-hydroxycyclohexyl) oxidanesulfonic acid Dextrin D-Ribose. *Anaerobiospirillum* may reduce fat deposition (Li et al., 2022), and studies have found that it is significantly enriched in the cecum of lean ducks compared to Peking ducks (Yang et al., 2022). *Lactobacillus* can improve the feed efficiency of chickens (Gao et al., 2017). These findings suggest that the gut microbial can regulate the feed efficiency of animals. Different gut microbial communities in different intestinal segments determines their different functions, and cecal microbiota has the greatest impact on RFI (Gao et al., 2017). Wen et al. found that the abundance of *Akkermansiamuciniphila*, *Parabacteroides*, *Lactobacillus,* and *Slackia* was significantly associated with RFI (Wen et al., 2021). We identified that *Phascolarctobaterium* and *Anaerobiospirillum* may further regulate RFI and improve feed utilization efficiency by producing SCFAs to provide energy for ABC transporter proteins.

## Conclusion

In summary, the production performance and cecal microflora composition of Shaoxing ducks and subsequently, their metabolome was analyzed. Difference analysis showed that FI and FCR were significantly higher in the group HRFI than in the LRFI. We found that cecal contents of ducks with better feed efficiency showed a diverse microbial community. However, as an important index, the relative abundance of *Firmicutes*, which is related to energy metabolism, was higher in the LRFI than in the HRFI group. We assessed 6,674 metabolites in all samples using the untargeted LC–MS approach and identified 338 significantly differentially expressed metabolites in rectal contents of the groups LRFI and HRFI. Integrated analysis of the microbiome and metabolome revealed 93 differentially expressed metabolites correlated with the relative abundance of 20 genera. We identified that *Phascolarctobaterium* and *Anaerobiospirillum* may further regulate RFI and improve feed utilization efficiency by producing SCFAs to provide energy for ABC transporter proteins. These results revealed the relationship between microbiome and metabonomics in laying ducks with different residual feed intake, and provided theoretical basis for further study on the relationship between them.

## Data availability statement

The datasets presented in this study can be found in online repositories. The names of the repository/repositories and accession number(s) can be found in the article/Supplementary material.

## Ethics statement

Animals used in this study were raised in accordance with the national standard of Laboratory Animal Guidelines for ethical review of animal welfare. All experiment procedures were approved by the Animal Use Committee of Zhejiang Academy of Agricultural Sciences (2022ZAASLA40). Written informed consent was obtained from the owners for the participation of their animals in this study.

## Author contributions

HS prepared the manuscript and collected some data. WX, TG, JS, CL, LC, YT, and GL collected the samples. LL and TZ were responsible for the design and direction of the experiment. All authors contributed to the article and approved the submitted version.

## Funding

This work was supported by Zhejiang Provincial Natural Science Foundation of China under Grant No. LZ23C170001, the National Key Research and Development Program of China (2022YFD1300100), the China Agriculture Research System of MOF and MARA (CARS-42) and Zhejiang Province Agricultural New Breed Breeding Major Science and Technology Special Project (2021C02068).

## Conflict of interest

CL was employed by Hubei Shendan Health Food Co., Ltd.

The remaining authors declare that the research was conducted in the absence of any commercial or financial relationships that could be construed as a potential conflict of interest.

## Publisher’s note

All claims expressed in this article are solely those of the authors and do not necessarily represent those of their affiliated organizations, or those of the publisher, the editors and the reviewers. Any product that may be evaluated in this article, or claim that may be made by its manufacturer, is not guaranteed or endorsed by the publisher.

## Footnotes

^1^ http://drive5.com/uchime/uchime_download.html

^2^ http://www.drive5.com/usearch/manual/uchime_algo.html

^3^ http://rdp.cme.msu.edu/index.jsp
