# Supplementary material for: Association of residual feed intake with intestinal microbiome and metabolome in laying period of ducks
Source: Front Microbiol. 2023 May 12;14:1138914. doi: 10.3389/fmicb.2023.1138914 (PMC10213451; doi:10.3389/fmicb.2023.1138914)
Supplement: Supplementary file 1 [file Data_Sheet_1.PDF]

Supplementary Materials

Frontiers in Microbiology

Association of residual feed intake with intestinal microbiome and metabolome in laying period of ducks

Hanxue SUN[1]. Wenwu XU[1]. Tiantian GU[1]. Jing SUN[2]. Chengfeng LI[3]. Li CHEN[1]. Yong TIAN[1]. Guoqin LI[1]. Lizhi LU[1*] . Tao Zeng[1*]

1. State Key Laboratory for Managing Biotic and Chemical Threats to the Quality and Safety of Agro-products; Key Laboratory of Livestock and Poultry Resources (Poultry) Evaluation and Utilization, Ministry of Agriculture and Rural Affairs; Institute of Animal Husbandry and Veterinary Medicine, Zhejiang Academy of Agricultural Sciences, Hangzhou, 310021, PR China
2 Institute of Animal Husbandry and Veterinary Medicine, Hubei Academy of Agricultural sciences, Wuhan, 430064, PR China
3 Hubei Shendan Health Food Co., Ltd., Xiaogan, 430000, PR China

Corresponding author：Lizhi LU and Tao Zeng;

E-mail address：lulz@zaas.ac.cn (L. L.); zengtao4009@126.com (T. Z.);

Telephone：13306813018 (L. L.); 18858290605 (T. Z.);

Fax numbers：0086571-86406682 (L. L.); 0086571-86404216 (T. Z.).

Table S1

Composition and main characteristics of the basal diet.

Ingredients	Content(g/kg)	Nutrient	Content(g/kg)
Maize grain	400	Metabolizable energy	11.2[b]
Wheat	290	Crude protein	16.5
Soybean meal	120	Total phosphorus	0.70
Wheat bran	90	Total calcium	3.35
Calcium hydrophosphate	12	Total lysine	0.79
Stone powder	80	Total methionine	0.40
Salt	3	Ether extract	29.0
Premix[a]	5		

[a]Supplied per kg of diet: vitamin A 1500 U, vitamin D_3 210 U, vitamin E 8 U, vitamin B_2 4 mg, vitamin B_5 8 mg, vitamin B_{12} 10 µg, vitamin B 0.5 mg, vitamin B_1 1.5 mg,vitamin B_6 3.0 mg, Fe 60 mg, Zn 35 mg, Mn 65 mg, Cu 6 mg, Se 0.4 mg.

[b]Unit: MJ/kg.

Table S2

Clean tag data statistics

Sample_name	Raw_PE	Qualified	Nochime	Base(nt)	AvgLen(nt)	Q20	GC%	Effective%
H1	54,269	47,560	42,595	17,846,354	419	88.14	49.94	78.49
H2	55,302	48,365	43,635	18,250,131	418	88.14	50.56	78.9
H3	53,159	45,840	40,694	17,113,168	421	88.41	50.2	76.55
H4	54,582	47,120	42,000	17,662,275	421	88.21	50.15	76.95
H5	53,098	45,947	41,698	17,510,804	420	87.94	51.32	78.53
H6	51,821	44,692	39,327	16,489,885	419	87.99	50.7	75.89
H7	53,141	46,472	41,625	17,452,947	419	87.59	50.64	78.33
H8	55,048	48,054	42,417	17,841,749	421	88.47	49.39	77.05
H9	54,894	47,883	41,574	17,429,281	419	87.83	50.1	75.74
H10	54,334	46,564	39,907	16,711,347	419	87.29	50.99	73.45
L1	51,321	44,636	40,495	16,953,336	419	88.27	50.61	78.91
L2	50,994	44,217	40,360	16,920,483	419	88.34	50.85	79.15
L3	52,117	44,626	39,074	16,387,775	419	88.08	50.43	74.97
L4	52,971	46,270	41,669	17,494,476	420	88.1	50.81	78.66
L5	54,029	45,873	42,283	17,674,897	418	88.42	51.43	78.26
L6	50,755	44,243	40,292	16,819,126	417	87.76	51.47	79.39
L7	52,826	45,701	41,680	17,479,706	419	88.3	51.01	78.9
L8	55,025	47,760	41,592	17,452,768	420	88.21	50.48	75.59
L9	53,515	46,657	41,949	17,639,174	420	88.3	50.11	78.39
L10	51,788	44,407	38,378	16,095,398	419	87.81	50.54	74.11

Table S3
The alpha diversity analysis index of different groups.

Sample	chao1	shannon	simpson	goods_coverage	Group
H1	749	6.845348042	0.977113361	0.995317073	HRFI
H2	404.1363636	5.143171765	0.939759842	0.996682927	HRFI
H3	606.893617	5.773388869	0.929542478	0.995668293	HRFI
H4	663.0491803	5.964813721	0.956689675	0.995317073	HRFI
H5	519.2380952	5.707069712	0.955724919	0.996058537	HRFI
H6	576.625	6.075943515	0.952038896	0.996136585	HRFI
H7	496.6666667	5.653801917	0.948869306	0.996643902	HRFI
H8	504.2758621	5.1265911	0.910833819	0.996214634	HRFI

H9	494.6612903	5.466900425	0.945883103	0.99617561	HRFI
H10	682.1666667	6.188537073	0.958303258	0.995121951	HRFI
L1	742.4375	6.627089799	0.974530734	0.994887805	LRFI
L2	755.3690476	7.085930201	0.979100116	0.995707317	LRFI
L3	710.1724138	6.936289531	0.981013567	0.995668293	LRFI
L4	635.8	6.745829373	0.977257915	0.996136585	LRFI
L5	591	6.471244864	0.976009801	0.996292683	LRFI
L6	737.6666667	7.101673091	0.983572955	0.995473171	LRFI
L7	688.038961	6.632147371	0.969007374	0.995785366	LRFI
L8	701.5147059	7.005016259	0.983151647	0.995395122	LRFI
L9	689.6428571	6.549624803	0.971144385	0.995707317	LRFI
L10	529.4516129	6.318127127	0.971335528	0.996058537	LRFI

The alpha diversity analysis index of different samples under 97% consistency threshold is statistically analyzed. Whether the species diversity difference between groups is significant is analyzed through T-test and wilcox rank sum test.

Table S4

Parameter values of principal component analysis (PCA) and orthogonal projections to latent structures-discriminant analysis (OPLS-DA) models used for the exprimental ducks.

Type	$R^2X(cum)$[1]	$R^2Y(cum)$[2]	$Q^2(cum)$[3]
PCA score plot	0.230		
OPLS-DA score plot	0.192	0.998	0.466

[1,2]$R^2Y(cum)$ and $R^2Y(cum)$ represent the interpretability of models.

[3]$Q^2(cum)$ represents the predictability of models.

Table S5

Identification of significantly different rectal metabolites of ducks between the LRFI and HRFIgroups.

adducts	Name	VIP[1]	FC[2]	*p*-value	MZ[3]	R.T[4](s)
**Negative**						
M+FA-H	Ginsenoside Rh7	1.257782	0.459149	0.026157	681.42238	7.722
M+FA-H	Mirificin	1.834781	0.511315	0.049397	593.15145	4.07
M-H	9(S)-HODE	2.065004	0.662569	0.03523	295.22796	9.039
M+FA-H	1,2-Anhydridoniveusin	1.508323	3.507137	0.033604	421.15149	4.424
M-H	2-Feruloyl-1-sinapoylgentiobiose	1.069765	0.295423	0.011907	723.2148	4.511
M+FA-H	20S-Hydroxycholest-1-en-3,16-dione	2.204438	0.675658	0.008001	459.31188	10.141

M+FA-H	PS(MonoMe(11,3)/MonoMe(13,5))	1.589989	0.442213	0.04813	940.55136	4.71
M+FA-H	Linocinnamarin	1.061787	0.548055	0.049718	385.11441	3.891
M+FA-H	Atrolactic acid	1.394652	3.227394	0.043748	165.05475	4.424
M+FA-H	Medicoside F	1.729324	0.467813	0.024947	973.50161	5.232
M+FA-H	alpha-Carboxy-delta-decalactone	1.671152	1.981202	0.049089	259.11872	4.511
M+FA-H	L-Pyridosine	1.320762	2.716459	0.045569	299.12488	2.904
M+FA-H	Durupcoside A	1.873965	0.491188	0.035299	971.48595	5.393
M-H	4-(2-Aminophenyl)-2,4-dioxobutanoic acid	1.394662	5.05143	0.049231	206.04527	2.398
M+FA-H	Tuberoside	1.117606	0.438784	0.044662	637.39572	9.74
2M-H	5'-Methylthioadenosine	2.355361	0.404411	0.010237	593.17253	3.804
M+FA-H	6-(3-ethenylphenoxy)-3,4,5-trihydroxyoxane-2-carboxylic acid	1.553187	0.412249	0.036178	341.0887	3.208
M+FA-H	Deoxyuridine	2.354048	1.545991	0.024906	273.07312	1.285
M-H2O-H	cis-2-Methylaconitate	1.036373	6.59832	0.017435	169.01334	3.668
M-H	Gluconolactone	1.146686	1.353081	0.045272	177.03964	1.266
M-H	({6-[5,7-dihydroxy-2-(4-hydroxyphenyl)-4-oxo-4H-chromen-6-yl]-3,4,5-trihydroxyoxan-2-yl}methoxy)sulfonic acid	2.928182	0.305943	0.016216	511.05537	4.026
M-H2O-H	N,N'-diacetylchitobiose	1.85795	1.78841	0.006992	423.16276	2.05
M-H	Kynurenic acid	1.05699	4.127004	0.044893	188.03457	3.556
M-H	(S)-a-Amino-2,5-dihydro-5-oxo-4-isoxazolepropanoic acid N2-glucoside	1.249528	1.99272	0.014842	333.09416	1.244
M-H	PGF2alpha dimethyl amide	2.488736	0.227286	0.020704	380.28094	9.881
M+FA-H	Carcinomedin	2.669515	0.51181	0.002174	473.32733	10.399
M+FA-H	Tyrosyl-Gamma-glutamate	2.408494	0.589351	0.028308	354.1307	3.019
M+FA-H	5-Hydroxyindoleacetaldehyde	1.050073	2.998652	0.031958	220.06115	4.07
M+FA-H	Cucurbitacin E	2.208226	0.491507	0.025124	601.30254	8.803
M-H	3,10-dihydroxydecanoic acid	1.760878	4.704962	0.018185	203.12827	4.466
M+FA-H	5-Tetradecenoic acid	2.244023	1.378694	0.034527	271.19175	7.84
M-H2O-H	6-O-(3R,4-dihydroxy-2-methylene-butanoyl)-beta-D-glucopyranose	1.309208	3.137876	0.034266	275.07758	2.95
M+FA-H	Alpha-Linolenoyl ethanolamide	2.479625	0.21339	0.019408	366.26499	9.694
M+FA-H	Tinctormine	2.017697	0.366281	0.030857	638.17292	3.302

M-H2O-H	10,11-dihydro-20-trihydroxy-leukotriene B4	1.790839	2.566661	0.020453	367.21257	4.555
M-H	N2-Succinyl-L-glutamic acid 5-semialdehyde	2.019635	2.467494	0.03033	230.06668	1.006
M-H	(1S,2S,4R,8S)-p-Menthane-1,2,8,9-tetrol 2-glucoside	1.479615	0.333267	0.039856	365.18157	4.138
M+FA-H	6-[2-carboxy-2-(hydroxymethyl)-2-methylethoxy]-3,4,5-trihydroxyoxane-2-carboxylic acid	1.899997	1.647352	0.043692	355.08959	1.486
2M-H	(-)-trans-3,4-Dihydro-4,8-dihydroxy-3-methyl-1H-2-benzopyran-1-one	1.365569	11.402857	0.021943	387.10897	5.028
M-H2O-H	3-(3,4-dihydroxyphenyl)-2-(sulfooxy)propanoic acid	1.04506	0.374546	0.008139	258.99189	3.668
M-H2O-H	L-DOPA 3'-glucoside	1.525311	8.25446	0.034997	340.10383	3.781
M-H	9-oxo capric acid	1.292694	2.592679	0.032058	185.11749	4.961
M+FA-H	Hypoglycin B	1.515997	2.656847	0.021436	315.12014	1.463
M-H2O-H, M-H	N-(gamma-Glutamyl)ethanolamine	2.079376	1.986914	0.040296	189.08737	0.924
M+FA-H	Deoxycytidine	1.402274	1.825869	0.017235	272.08899	1.285
M+FA-H	5-Hepteneoylglycine	1.54039	0.558899	0.006667	230.10308	4.268
M-H	4,4''-bis(N-feruloyl)serotonin	1.665831	0.311702	0.016537	701.26371	9.18
2M-H	7-Methylinosine	2.053875	1.802109	0.03307	565.19987	3.441
M-H2O-H	2-(3,4-dihydroxyphenyl)-4-[3,5,7-trihydroxy-2-(4-hydroxy-3-methoxyphenyl)-3,4-dihydro-2H-1-benzopyran-8-yl]-6-[3,4,5-trihydroxy-6-(hydroxymethyl)oxan-2-yl]-3,4-dihydro-2H-1-benzopyran-3,5,7-triol	2.226686	0.281325	0.020253	735.18932	3.914
M+FA-H	Saringosterol 3-glucoside	2.218225	0.391697	0.017901	635.41649	9.531
M+FA-H	4-(4-Hydroxyphenyl)-2-butanone	1.731506	9.307894	0.027235	209.08133	4.598
M+FA-H	12-hydroxyheptadecanoic acid	1.251052	2.651511	0.018412	331.24906	5.784
M-H2O-H, M-H	3,5,6-Trihydroxy-5-(hydroxymethyl)-2-methoxy-2-cyclohexen-1-one	1.538535	3.880838	0.038271	203.0556	2.904
M+FA-H	4-ethylphenylsulfate	1.344357	0.103553	0.043896	247.02802	5.255
M+FA-H	6-(3-ethylphenoxy)-3,4,5-trihydroxyoxane-2-carboxylic acid	2.452527	0.373002	0.012722	343.10399	3.277
M-H, M+FA-H	Araloside A	1.352322	0.526507	0.04581	925.48099	5.6
M+FA-H	2-Aminoheptanedioic acid	1.516412	2.006114	0.009646	220.08231	1.244
M+FA-H	5'-Hydroxy-3'-methoxysativan	1.67762	2.599983	0.024366	377.12487	4.466
M-H2O-H	Spinosin C	1.900357	0.434077	0.010445	735.18951	3.373
M-H	(3b,9R)-5-Megastigmene-3,9-diol 9-[apiosyl-(1->6)-glucoside]	1.261177	0.453599	0.017035	505.26585	5.463
M-H2O-H	Succinylacetoacetate	1.555194	2.36028	0.010475	183.02912	2.239

M-H2O-H, M-H	Isomugineic acid	1.397112	1.856951	0.037558	319.11474	0.985
M+FA-H	Propyl 2-furanacrylate	1.67851	3.349571	0.037056	225.07647	3.623
2M-H	1,2-Dihydroxy-3-keto-5-methylthiopentene	1.139867	2.945499	0.036375	323.06291	4.4
M-H	Isopetasoside	1.872676	5.013277	0.026614	395.20781	5.005
M-H	5-Sulfoxymethylfurfural	1.211347	0.114921	0.046717	204.98071	1.509
2M-H	2-Hydroxyvaleric acid	1.622675	3.262555	0.020882	235.11862	1.767
M+FA-H	(22S)-1alpha,22,25-trihydroxy-23,24-tetradehydro-24a-homo-20-epivitamin D3 / (22S)-1alpha,22,25-trihydroxy-23,24-tetradehydro-24a-homo-20-epicholecalciferol	1.208166	4.763903	0.0345	487.30665	6.629
M+FA-H	Lenalidomide	1.584551	4.417472	0.031992	304.09437	2.239
2M-H	Sulfate	1.165311	0.153516	0.047597	194.92682	1.09
M-H	Undecylenic acid	1.746863	3.557356	0.04827	183.13822	5.784
M+FA-H	Hydroxyphenylacetylglycine	2.39248	1.90977	0.014861	254.06702	1.838
M-H	1-(1,2,3,4,5-Pentahydroxypent-1-yl)-1,2,3,4-tetrahydro-beta-carboline-3-carboxylate	1.276688	3.525272	0.042215	365.13573	3.781
M+FA-H	Cysteinyl-Hydroxyproline	1.088794	2.279904	0.022477	279.0657	2.121
M-H	(2-hydroxy-5-{3,5,7-trihydroxy-6-[3,4,5-trihydroxy-6-(hydroxymethyl)oxan-2-yl]-3,4-dihydro-2H-1-benzopyran-2-yl}phenyl)oxidanesulfonic acid	1.042867	0.109571	0.023521	531.0817	2.811
M-H2O-H	2-{[hydroxy(2,3,4,5-tetrahydroxyphenyl)methylidene]amino}acetic acid	1.109679	3.165095	0.025009	224.01971	3.579
M+FA-H	Muricin A	1.399873	0.523104	0.027652	641.46347	8.076
M-H, M+FA-H	28-Glucosyloleanolic acid 3-[arabinosyl-(1->2)-6-methylglucuronide]	1.697889	0.51701	0.010497	939.49658	7.257
M-H	{4-[3-(5-hydroxy-2,2-dimethyl-2H-chromen-6-yl)prop-2-enoyl]phenyl}oxidanesulfonic acid	1.475866	0.254	0.000483	401.06964	2.003
M-H2O-H	Prostaglandin D2	2.478707	2.952941	0.049417	333.20729	6.371
M+FA-H	Sonchuionoside C	1.952551	0.621268	0.024371	431.19217	4.049
M+FA-H	N-Methylcalystegine B2	2.673987	1.805984	0.003179	234.09809	1.373
M+FA-H	Isofucosterol glucoside	1.667944	0.420148	0.037301	619.42168	12.16
M-H	(gamma-Glutamyl-gamma-glutamyl)-S-methylcysteine	1.924299	1.554394	0.036483	392.11281	2.835
M-H2O-H	Methotrexate	2.05882	0.495235	0.021427	435.15565	4.443
M+FA-H	[4-(1-hydroxy-3-oxobutyl)-2-methoxyphenyl]oxidanesulfonic acid	1.995806	0.058491	0.015743	335.04451	3.324
M-H2O-H, M+FA-H	Doxepin N-oxide glucuronide	1.916056	0.509339	0.01664	517.19302	4.489
M-H2O-H, M+FA-H	LysoPA(8:0/0:0)	1.477995	2.656675	0.031258	343.11489	3.254

2M-H	4-Hydroxyproline	1.743051	1.669715	0.004975	261.10922	0.945
M+FA-H	beta-Sitosterol 3-O-beta-D-galactopyranoside	1.537699	0.512936	0.018888	621.43737	13.031
M+FA-H	Campesteryl glucoside	1.652586	0.476578	0.026209	607.4219	12.615
M+FA-H	N-Acetylvanilalanine	1.542125	4.560405	0.044587	298.0934	3.982
M-H2O-H	Glucosinalbin	1.261536	0.209522	0.001342	406.02712	4.003
2M-H	5-Hydroxyomeprazole	1.606355	0.315355	0.043357	721.2141	5.347
M-H2O-H	Lucuminoside	2.045486	3.562056	0.049698	408.13041	3.869
M+FA-H	N-Carbamoyl-2-amino-2-(4-hydroxyphenyl)acetic acid	1.499637	1.704361	0.01764	255.06237	2.05
M-H2O-H, 2M-H	Histidinyl-Methionine	1.420434	3.317071	0.030332	267.0911	4.687
2M-H	D-Glyceraldehyde 3-phosphate	2.585065	1.487392	0.019932	338.98867	7.304
M+FA-H	(3beta,5alpha,9alpha,22E,24R)-5,9-Epidioxy-3-hydroxyergosta-7,22-dien-6-one	1.189773	5.156805	0.030111	487.30655	7.35
2M-H	Aesculetin	1.670537	0.160217	0.022828	355.04551	1.307
M+FA-H	Benzyl methyl sulfide	1.807387	1.840077	0.044664	183.04848	1.395
2M-H	L-Aspartic acid	1.156965	2.172189	0.040088	265.06786	1.58
M+FA-H	Dihydroroseoside	2.204265	0.382467	0.009804	433.20813	4.003
2M-H	L-Proline	2.149698	2.040986	0.00642	229.11911	3.937
M-H, 2M-H	Toyocamycin	2.08259	1.534107	0.030019	290.08833	1.244
2M-H	N-Methylnicotinamide	1.160671	11.755136	0.039747	271.11911	2.811
M+FA-H	Tyrosyl-Cysteine	2.034783	11.313523	0.029479	329.08104	3.645
M+FA-H	(S)-5-Diphosphomevalonic acid	1.102067	1.292254	0.040878	353.0044	7.327
2M-H	Norsanguinarine	1.768408	0.211837	0.040656	633.12875	0.886
M-H2O-H	{5-[(E)-2-{3,5-dihydroxy-4-[(1E)-3-methylbuta-1,3-dien-1-yl]phenyl}ethenyl]-2-hydroxyphenyl}oxidanesulfonic acid	1.889644	0.364923	0.011085	371.05905	2.858
M+FA-H	PC(10:0/4:0)	1.640545	0.13026	0.006934	526.27833	4.893
M-H, M+FA-H	Malonylcarnitine	1.784273	3.840095	0.024018	292.1039	0.985
M+FA-H	Gamma-Tocotrienol	1.537549	4.131245	0.015626	455.31696	8.425
M+FA-H	6-{[10-butanoyl-3-hydroxy-6-(2-hydroxypropyl)-2,2-dimethyl-8-oxo-2H,3H,4H,8H-pyrano[3,2-g]chromen-5-yl]oxy}-3,4,5-trihydroxyoxane-2-carboxylic acid	1.324005	0.292312	0.006339	611.19816	4.224
M-H2O-H	PA(16:1(9Z)/14:1(9Z))	2.031515	0.354337	0.015526	597.39312	12.615

M-H2O-H	Sambacin	1.533936	0.630828	0.011435	521.2037	4.314
2M-H	S-Isopropyl 3-methylbut-2-enethioate	1.7462	5.944116	0.028235	315.14523	4.443
M+FA-H	3,4,5-trihydroxy-6-{[8-(2-hydroxypropan-2-yl)-2-oxo-4-propyl-2H,8H,9H-furo[2,3-h]chromen-5-yl]oxy}oxane-2-carboxylic acid	1.497108	0.196005	0.016787	525.15956	3.645
M+FA-H	UK-121,265	1.598591	0.255363	0.048862	410.10636	4.026
2M-H	Donepezil metabolite M4	1.309346	0.342887	0.022747	577.32598	9.904
M+FA-H	D-glycero-L-galacto-Octulose	2.018718	2.380217	0.03365	285.08339	0.945
M-H2O-H	PA(a-13:0/18:2(9Z,11Z))	1.524841	0.406705	0.023376	611.40859	13.035
M-H2O-H, M-H	2-(1,2,3,4-Tetrahydroxybutyl)-6-(2,3,4-trihydroxybutyl)pyrazine	1.787596	2.641145	0.00258	303.11988	0.985
M+FA-H	Phloretin xylosyl-galactoside	1.858079	0.090511	0.011279	613.17764	1.744
M+FA-H	Carbimazole	1.938074	3.44951	0.022723	231.04419	4.224
M-H	2-(4-Hydroxyphenyl)naphthalic anhydride	1.141259	2.571819	0.021136	289.05011	2.026
M-H	(±)-Flufenprox	1.521917	0.356442	0.040358	449.11254	2.332
M+FA-H	{11-hydroxy-16,18-dioxo-6,8,19-trioxapentacyclo$[10.7.0.0^{2,9}.0^{3,7}.0^{13,17}]$nonadeca-1(12),2(9),4,10,13(17)-pentaen-14-yl}oxidanesulfonic acid	2.217267	0.24983	0.003825	438.99744	4.424
M-H2O-H	Anthenoside A	1.815372	0.515312	0.024854	660.44854	13.031
M-H2O-H	3,5-di-O-(beta-Glucopyranosyl) pelargonidin 6''-O-4, 6'''-O-1-cyclic malate	2.88368	0.282854	0.034587	674.14966	3.302
M+FA-H	Ganosporeric acid A	2.181812	0.644242	0.023198	571.25662	5.531
M-H2O-H	2-(acetylamino)-1,5-anhydro-2-deoxy-4-O-b-D-galactopyranosyl-D-arabino-Hex-1-enitol	1.689153	4.727048	0.030609	346.11561	3.759
M+FA-H	3-(3,4,5-trihydroxyphenyl)propanoic acid	1.062845	1.525806	0.023934	243.05091	1.603
M-H	3,4,5-trihydroxy-6-{4-[(1E)-3-oxo-3-[(3,4,5,6-tetrahydroxyoxan-2-yl)methoxy]prop-1-en-1-yl]phenoxy}oxane-2-carboxylic acid	1.403961	0.282227	0.007814	501.12566	3.277
M+FA-H	3,5-Dihydroxyphenyl 1-O-(6-O-galloyl-beta-D-glucopyranoside)	2.019001	0.048083	0.036486	485.09162	0.886
M-H2O-H	[4-(5,7-dihydroxy-6,8-dimethyl-4-oxo-3,4-dihydro-2H-1-benzopyran-2-yl)phenyl]oxidanesulfonic acid	1.555091	0.1686	0.038415	361.03813	1.557
M-H	Pentoxifylline	1.569981	5.795657	0.018981	277.12939	1.627
M+FA-H	Queuine	1.563781	2.734285	0.040128	322.11702	13.439
M-H	Methionyl-Methionine	1.113996	0.514894	0.004922	279.08471	3.111
M+FA-H	2-Methoxyestrone 3-sulfate	2.010755	4.023433	0.033289	425.12792	5.255
M+FA-H	2-amino-4-[(2-{[2-carboxy-2-hydroxy-1-(4-hydroxyphenyl)ethyl]sulfanyl}-1-[(carboxymethyl)-C-hydroxycarbonimidoyl]ethyl)-C-hydroxycarbonimidoyl]butanoic acid	1.954187	6.024318	0.028139	532.12629	6.211
2M-H	Alpha-T	2.052623	0.152635	0.025486	330.97403	1.07

M-H	L-4-Chlorotryptophan	1.677501	2.796584	0.046392	237.0437	2.216
2M-H	Riboflavin reduced	1.626778	0.09263	0.016406	695.20456	3.348
M+FA-H	2-amino-4-({1-[(carboxymethyl)-C-hydroxycarbonimidoyl]-2-[(2-methyl-3-oxo-1-phenylpropyl)sulfanyl]ethyl}-C-hydroxycarbonimidoyl)butanoic acid	2.19516	5.676696	0.022874	498.15523	3.602
2M-H	Lignoceroyl-EA	1.495394	0.279264	0.041434	821.80912	4.576
M+FA-H	5-Taurinomethyl-2-thiouridine	2.0158	0.265888	0.0064	442.06012	1.35
M-H2O-H	Cefdinir	1.657814	0.192755	0.025318	376.01678	3.982
M-H	S-Adenosylhomocysteine-d4	1.758057	2.509293	0.02817	387.14134	2.026
M-H	S-methylazathioprine	1.502567	2.076244	0.048887	291.0548	1.58
M-H2O-H	Kaempferol 7-(6''-galloylglucoside)	2.127495	0.329262	0.020215	581.09321	1.885
M+FA-H	({6-[5,7-dihydroxy-2-(4-methoxyphenyl)-4-oxo-4H-chromen-6-yl]-3,4,5-trihydroxyoxan-2-yl}methoxy)sulfonic acid	1.608511	0.103103	0.030221	571.07665	4.182
2M-H	E-3174	1.50568	0.29129	0.039748	871.27274	3.826
M-H2O-H	PG(20:1(11Z)/0:0)	1.742366	2.826941	0.015591	519.3066	6.696
2M-H	Trazodone	2.871876	2.557523	0.001445	741.29512	4.291
M+FA-H	Diazoxide	1.73274	0.296292	0.003682	274.99025	1.026
M-H2O-H	Leonoside A	1.409244	0.158223	0.023505	751.24577	4.049
M-H, M+FA-H	Demonomethylchlorpromazine	2.057965	1.619434	0.041817	303.07248	1.744
**Positive**						
M+H	Genistin	1.28435	0.242309	0.029431	433.11312	4.347
M+H	Daidzin	1.139885	0.239861	0.019323	417.11782	4.079
M+H, M+Na	Sandosaponin B	2.184596	0.505581	0.011616	957.50088	7.103
M+H	Soyasaponin IV	1.82909	0.574078	0.037894	767.45492	7.035
M+H-H2O	Ginsenoside Mc	1.461991	0.539014	0.036435	737.48106	6.879
M+H, M+K, M+NH4, M+Na, M+H-H2O	Soyasaponin II	1.745978	0.569582	0.044452	913.51171	6.879
M+H-H2O	1alpha,25-dihydroxy-26,27-dimethyl-20,21-didehydro-23-oxavitamin D3 / 1alpha,25-dihydroxy-26,27-dimethyl-20,21-didehydro-23-oxacholecalciferol	1.70328	0.484346	0.01564	429.33657	9.804
M+H, M+K	Aspartyl-Valine	2.342204	0.484669	0.013572	233.11303	1.392
M+H	C16 Sphinganine	2.16708	0.593317	0.027351	274.27361	6.526
M+NH4, M+Na, M+H	Cynaroside A	1.359166	0.403298	0.018619	462.23315	3.653

M+Na	PKHdiA-PE	1.553613	0.452105	0.006837	630.3008	4.12
M+H-H2O, M+H	(-)-11-hydroxy-9,10-dihydrojasmonic acid	1.008607	3.390849	0.040951	211.13278	4.306
M+H	Corey PG-Lactone Diol	1.324595	5.478178	0.024128	269.17452	4.079
M+NH4, M+Na	17-hydroxy stearic acid	1.945569	0.644582	0.045969	318.2999	6.593
M+H-H2O, 2M+H, M+H	Humulenol I	1.590436	0.559718	0.034994	441.37231	6.879
M+H	Apiin	1.301628	0.417759	0.020014	565.15486	4.243
M+H	9S-hydroxy-12R,13S-epoxy-10E,15Z-octadecadienoic acid	1.163797	1.467534	0.037319	311.22139	5.64
M+H	Aspartyl-Isoleucine	1.833839	0.529174	0.031178	247.12862	2.492
M+H	Threoninyl-Isoleucine	1.363342	2.216622	0.031295	233.14937	2.692
M+H	Quercetin 3-(6''-(E)-sinapoylsophoroside)-7-rhamnoside	2.714718	0.264538	0.0174	979.26741	3.797
M+H	Apigenin 7-allosyl-(1->2)-glucoside	1.336364	0.461122	0.011497	595.16503	4.079
M+H-H2O, M+H	alpha,gamma-Onoceradienedione	1.568625	0.60653	0.010186	439.35684	7.26
M+H, M+Na	Methyl 1-methoxy-1H-indole-3-carboxylate	1.301781	0.384111	0.00176	206.08099	4.866
M+NH4	Absinthin	1.365899	3.364989	0.032193	514.31688	8.481
M+H-H2O	6-[4-(5,7-dihydroxy-4-oxo-3,4-dihydro-2H-1-benzopyran-2-yl)phenoxy]-3,4,5-trihydroxyoxane-2-carboxylic acid	1.859706	0.152344	0.041321	431.09709	4.079
M+NH4	(+)-Marmasmic acid	1.838677	0.514256	0.02528	280.1542	3.856
M+H	alpha-Hydrojuglone 4-O-b-D-glucoside	1.275439	0.392112	0.024919	339.10724	3.978
M+H-H2O, M+Na, M+H	Ikarisoside D	1.889607	2.262295	0.046144	525.17552	4.474
M+H, M+Na	Saccharopine	1.309618	1.64841	0.049181	277.13933	0.959
M+H-H2O, M+H	1-O-Sinapoyl-β-D-glucose	2.100218	0.344867	0.007718	369.11796	4.453
M+H-H2O, M+H	Lysyl-Proline	1.938813	1.728112	0.03017	226.15483	1.296
M+H-H2O, M+H	N-(3-carboxypropanoyl)-N-hydroxycadaverine	2.495641	1.93984	0.033121	219.13386	0.959
M+Na	5alpha-cholestan-3alpha,12alpha,16alpha-triol	1.27389	2.526321	0.043737	443.34815	5.343
M+Na, 2M+Na, M+K, M+NH4	3alpha,12alpha-Dihydroxy-15-oxo-5beta,14beta-cholan-24-oic Acid	2.055884	2.78684	0.043133	424.30539	6.858
M+H, 2M+H	Prolyl-Glutamine	1.687212	1.480359	0.018669	244.1291	0.959
M+H-H2O	1-[(5-Amino-5-carboxypentyl)amino]-1-deoxyfructose	1.619953	2.342652	0.040644	291.15493	1.261
M+H-H2O	Ichangin 4-glucoside	1.675268	4.837425	0.023392	633.25482	4.432

M+H	4-Hydroxy-6-methyl-3-(1-oxobutyl)-2H-pyran-2-one	1.100955	0.394026	0.029703	197.08067	3.896
2M+H	2-(2,6-dihydroxy-3,4-dimethoxycyclohexylidene)acetonitrile	1.460725	0.628411	0.036914	427.2077	3.592
M+NH4	N2-Maltulosylarginine	1.684053	0.528828	0.008901	516.24964	4.264
M+NH4	(E)-Casimiroedine	1.049102	1.867137	0.029008	435.22387	3.407
M+H	Cyanin	2.049665	0.234516	0.009667	611.15978	3.797
M+H	Aspartyl-Tryptophan	1.131214	0.522535	0.012015	320.12358	3.877
M+NH4, M+Na	10-Hydroxy-octadec-12Z-enoate-9-beta-D-glucuronide	1.825947	0.440551	0.029007	508.31153	6.413
M+H	Threoninyl-Phenylalanine	2.331442	2.101629	0.023517	267.13378	3.468
M+H, M+NH4, M+H-H2O	Pyridoxine (Vitamin B6)	1.624633	2.91615	0.03295	187.10763	1.226
M+H	L-alpha-Amino-1H-pyrrole-1-hexanoic acid	1.158381	1.695599	0.044281	197.12837	2.318
M+H	Pyridoxamine	1.394507	1.691463	0.040023	169.09709	1.352
M+H	Aspartyl-Tyrosine	1.799305	0.520915	0.020575	297.10809	1.666
M+Na	Pteroside Z	1.699597	0.435813	0.023071	417.18656	0.959
M+NH4	Pondaplin	1.277294	0.149118	0.026816	248.1279	6.458
2M+H	8-tetradecynoic acid	1.04363	0.463142	0.035792	449.36228	8.948
M+H	Aspartyl-Threonine	1.547585	0.707578	0.049052	235.09241	0.997
M+NH4	Carissanol	1.516833	5.196556	0.041566	394.18595	4.756
M+K	25-O-Desacetyl rifabutin	1.721934	0.391197	0.029322	819.35697	5.503
M+H	Glutaminyltyrosine	1.128095	2.338269	0.033252	310.13986	1.492
M+NH4, M+Na	Isoleucyl-Threonine	1.554781	2.033155	0.049226	255.1338	3.387
M+H-H2O, M+Na	MG(10:0/0:0/0:0)	1.548604	1.458651	0.04287	269.17465	14.884
M+H-H2O, M+Na	3,4,5-trihydroxy-6-(3-{3,5,7-trihydroxy-8-[3,5,7-trihydroxy-2-(3-hydroxyphenyl)-6-[3,4,5-trihydroxy-6-(hydroxymethyl)oxan-2-yl]-3,4-dihydro-2H-1-benzopyran-4-yl]-3,4-dihydro-2H-1-benzopyran-2-yl}phenoxy)oxane-2-carboxylic acid	1.52718	0.270061	0.038826	867.23469	0.923
2M+H	5-Amino-2,3-dihydro-6-(3-hydroxy-4-methoxy-1-oxobutyl)-2,2-dimethyl-4H-1-benzopyran-4-one	1.023346	0.452067	0.043944	615.29016	7.442
M+H	Cinncassiol C3	1.153087	2.180364	0.021439	383.20535	4.646
M+K	Maltotriose	2.131463	0.336621	0.034063	543.13238	0.923
M+H	Threoninyl-Tyrosine	1.76132	2.007004	0.026381	283.12886	1.557

M+H	Xanthine	1.828093	3.067263	0.002298	153.04061	1.226
M+H-H2O, M+NH4	Tryptophyl-Alanine	1.732402	0.362752	0.021753	293.16042	3.468
M+H	N-palmitoyl glutamic acid	1.037847	0.464776	0.036655	386.2896	9.967
M+H-H2O	Hydrocotarnine	1.326689	0.517884	0.026192	204.10193	3.21
M+H-H2O	Homocitrulline	1.785008	1.636105	0.049491	172.10789	1.279
M+H-H2O	Glaucasterol	2.015581	0.772465	0.036053	365.3197	9.711
M+H	Stizolobate	1.872634	2.202066	0.030284	228.05007	2.274
M+Na	Avenestergenin B2	1.119343	5.994738	0.048604	615.36363	7.672
M+Na, M+K	3,4,5-trihydroxy-6-[(4-oxo-2-phenyl-4H-chromen-3-yl)oxy]oxane-2-carboxylic acid	1.418271	5.363939	0.040803	453.06032	2.429
M+H-H2O	Hv-NCC-1	2.344398	0.125907	0.021986	661.28562	8.433
M+H-H2O	5,10-Methylene-THF	1.641661	0.52397	0.036506	440.1685	1.226
M+H-H2O, M+K	Prolyl-Asparagine	1.758719	2.033087	0.030479	212.10291	0.959
2M+NH4	Moupinamide	1.564701	0.387692	0.006909	644.29594	5.274
M+H	9R-hydroxy-10E,12E-octadecadienoic acid, methyl ester	2.110721	1.705239	0.029757	311.25772	9.203
2M+K	Cynaratriol	2.612543	0.276815	0.00741	603.2547	4.367
2M+K	(R)-1-O-b-D-glucopyranosyl-1,3-octanediol	1.064702	0.417606	0.013354	655.33298	3.613
M+H	(1Z)-2-hydroxy-1-(4-hydroxy-3-methoxyphenyl)-7-(3-methoxyphenyl)hept-1-ene-3,5-dione	1.069879	0.494213	0.037317	371.1494	5.16
M+H	PG(15:1(9Z)/0:0)	1.786201	0.473223	0.037884	469.25613	6.503
M+H-H2O, 2M+NH4	Hydroferulic acid	1.070754	0.604586	0.020536	179.07004	2.005
M+Na	PA(18:0/22:5(7Z,10Z,13Z,16Z,19Z))	2.124137	1.480292	0.040773	773.51043	4.889
M+K, 2M+NH4	Arginyl-Threonine	1.224006	3.391201	0.047781	314.1238	3.632
M+H-H2O	(22E,24E)-1alpha,25-dihydroxy-22,23,24,24a-tetradehydro-24a,24b-dihomovitamin D3 / (22E,24E)-1alpha,25-dihydroxy-22,23,24,24a-tetradehydro-24a,24b-dihomocholecalciferol	1.329904	0.499394	0.010508	423.32537	9.295
M+H-H2O	OON-PA	1.089312	0.407047	0.012559	573.35366	9.363
M+NH4, 2M+H	N1-(2,4-Dimethoxybenzyl)-n2-(2-(pyridin-2-yl) ethyl)oxalamide	1.065645	3.015041	0.033348	361.1866	4.285
M+H	Moschamine	2.312968	0.575217	0.008225	353.14923	4.844
M+NH4, 2M+H	Salsoline-1-carboxylate	1.042514	1.70496	0.032064	255.13387	1.226
M+H-H2O	Spinochalcone B	2.2549	0.274989	0.000035	357.1845	3.572

M+K	1-Fluoro-25-hydroxy-16-ene-23-yne-26,27-hexadeuterovitamin-D3	1.200755	0.406811	0.023667	457.2795	6.99
M+NH4	PC(5:0/5:0)	1.26267	4.147471	0.038074	443.25317	4.56
M+K	PC(7:0/0:0)	2.053785	0.643155	0.022511	408.15502	6.503
M+H-H2O	N1,N10-Diferuloylspermidine	2.232483	0.406688	0.012391	480.2491	4.646
M+H	L,L-Cyclo(leucylprolyl)	1.917279	1.340039	0.036414	211.14395	14.75
M+H	3,4,5-trihydroxy-6-oxane-2-carboxylic acid	1.319509	0.494905	0.029576	415.08707	4.516
2M+K	N,N-Didesmethyltramadol	1.724007	0.461401	0.006559	509.27614	3.302
M+H, M+Na	Diacetolol	1.072471	2.177229	0.041269	309.18064	4.079
2M+NH4	1-Nonene	1.14995	0.631308	0.026715	270.31531	8.879
M+H	Iso-Olomoucine	2.397338	2.487829	0.015219	299.16004	1.333
2M+H	2,3,6-Trimethylphenol	1.330394	0.457426	0.04758	273.18466	8.017
2M+NH4	Strobilurin A	1.59306	0.386914	0.011081	534.28477	9.943
M+K	Galactosylglycerol	1.851251	0.300126	0.032262	293.06322	0.923
M+H, M+K	Boc-DVal(NMe)-Val-OMe	1.05009	1.927044	0.031486	345.23825	3.302
2M+NH4	IdoA2S-beta-1,4-GlcNAc(3S)-alpha-1,4-GlcA-beta-1,3-Gal-beta-1,3-Gal-beta-1,4-Xylose	2.683506	0.129013	0.030655	376.10589	3.322
2M+H	2-Ethoxy-3-methylpyrazine	1.294882	0.35419	0.020109	277.16583	3.714
2M+K	1,2,5-Trimethyl-1H-pyrrole	2.397797	0.562194	0.006402	257.14096	8.739
M+K	Armillaribin	2.649206	0.599747	0.005507	437.17061	4.432
2M+Na	Ascladiol	1.13813	0.162341	0.017653	335.07352	1.666
M+K	2beta,3alpha,7alpha,12alpha-Tetrahydroxy-5beta-cholestan-26-oic acid	1.005409	0.385039	0.03178	505.29289	10.503
2M+NH4	Isopentyl beta-D-glucoside	1.155437	0.343296	0.028221	518.31781	4.098
2M+NH4	3-(6,7-dimethoxy-2H-1,3-benzodioxol-5-yl)-3-hydroxypropanal	2.34123	0.193557	0.034192	526.19096	6.051
M+NH4	Arjunolic acid 3-glucoside	1.097887	0.372104	0.011544	668.43554	9.804
M+Na, 2M+NH4	1-(6Z,9Z,12Z,15Z-octadecatetraenoyl)-glycero-3-phosphate	1.175518	0.478914	0.028681	453.2017	4.712
M+H	L-Octanoylcarnitine	1.069423	2.425067	0.038702	288.21669	5.137
2M+Na	Dihydrothymine	1.861111	0.512729	0.036195	279.10705	6.879
M+Na	PHOOA-PG	1.26403	0.320821	0.010138	661.33346	9.804
2M+NH4	2-Hydroxypyridine	1.763619	3.715335	0.039454	208.10798	1.392

M+H	Sanaganone	1.165219	0.367885	0.031345	345.11131	3.428
M+H, 2M+Na	1-(4-Hydroxy-3-methoxyphenyl)-3-decanone	1.631754	2.573562	0.019318	579.35018	5.366
M+NH4	5,7alpha-Dihydro-1,4,4,7a-tetramethyl-4H-indene	1.026727	1.678382	0.006349	192.17475	13.406
2M+K	2-Amino-3,4-dihydroxypentanedioic acid	1.542284	0.120999	0.044474	397.04751	2.981
M+H-H2O	hesperetin-7-O-glucuronide	1.221124	0.227152	0.042609	449.10762	3.49
M+NH4	EI-1511-5	1.19028	0.506037	0.03827	538.25456	4.623
M+H-H2O	PI(22:4(7Z,10Z,13Z,16Z)/0:0)	1.852097	0.243655	0.041968	631.32451	5.001
2M+K	Pargyline	1.363922	0.08922	0.049416	357.17134	5.297
M+NH4	Lisdexamfetamine	2.952499	0.465072	0.028557	281.23347	5.434
M+Na	S-Adenosylmethioninamine	1.322793	0.522424	0.02551	378.14459	7.511
2M+NH4	gamma-Glutamyl-beta-(isoxazolin-5-on-2-yl)alanine	1.011471	0.296068	0.024579	620.21842	4.389
M+Na	CD 1790	1.073562	0.332552	0.042139	496.20768	7.216
M+NH4	LysoPA(24:1(15Z)/0:0)	1.097622	0.303812	0.035542	538.38534	7.834
M+H	beta-D-Glucopyranosyl-11-hydroxyjasmonic acid	1.654082	1.705672	0.048189	389.18183	2.582
2M+Na	Dehydroanonaine	1.236775	0.191324	0.016328	549.18101	3.714
M+Na	Arginyl-Methionine	1.965616	2.376673	0.031444	328.14142	6.276
M+H	Asclepin	1.647097	2.464986	0.045437	575.28605	5.092
M+K	(5E)-(24R)-24,25-dihydroxy-[6,19,19-trideutrio]vitamin D3 / (5E)-(24R)-24,25-dihydroxy-[6,19,19-trideutrio]cholecalciferol	1.028998	5.439675	0.019793	458.31098	7.126
M+NH4	3-O-alpha-L-rhamnopyranosyl-3-hydroxydecanoyl-3-hydroxydecanoic acid	2.305153	0.299868	0.013178	522.36339	7.488
M+H-H2O, M+H	6,8a-Seco-6,8a-deoxy-5-oxoavermectin ''2a'' aglycone	1.236569	0.416165	0.018196	587.35685	9.804
2M+Na	Portulacaxanthin II	1.032027	0.328898	0.012186	771.21094	4.325
M+NH4	(6RS)-22-oxo-23,24,25,26,27-pentanorvitamin D3 6,19-sulfur dioxide adduct	1.398086	0.285718	0.023408	410.23586	8.017
M+NH4, 2M+NH4	5,8,11-dodecatriynoic acid	1.87997	2.261868	0.048898	394.19708	3.613
M+H	Nicardipine	1.059238	5.817269	0.042681	480.21273	5.069
M+H	Capryloylglycine	1.748834	4.275815	0.025881	202.1437	1.314
2M+Na	1-(2-Thienyl)-1-butanone	1.115945	0.393377	0.027758	331.0809	4.911
M+H-H2O	Resveratrol 4'-(6-galloylglucoside)	1.862291	0.445499	0.01083	525.13899	5.848
M+H	Kaempferol 3-rhamnoside 7-galacturonide	1.93351	0.445835	0.028591	609.1446	4.285

M+K	Aminopterin	2.434657	0.243112	0.017162	479.1187	4.453
M+K	Isothipendyl	1.759019	2.159882	0.021513	324.09328	4.602
M+NH4	Erucic acid	1.515933	0.371268	0.040136	356.35207	8.925
M+K	PS(14:0/12:0)	1.384193	0.281011	0.014611	690.3762	9.781
M+H-H2O	All-trans-13,14-dihydroretinol	1.803872	0.451258	0.049678	271.24181	7.488
2M+NH4	2-hydroxymexiletine	1.119389	1.627727	0.035778	408.28539	9.479
M+K	Narceine	1.561935	0.061792	0.033986	484.13857	2.827
M+H	Italidipyrone	2.307262	0.156672	0.027695	543.22367	9.479
M+NH4	3-hydroxyarachidonoylcarnitine	1.293456	4.175752	0.021751	481.36364	5.757
2M+Na	Sulfinpyrazone	1.491893	0.387282	0.039667	831.23193	4.602
2M+H	2-Heptyl-4,5-dimethylthiazole	1.458763	1.887902	0.015838	423.28463	5.711
M+H, 2M+Na	(2R,3R,4R)-2-Amino-4-hydroxy-3-methylpentanoic acid	1.540592	2.432616	0.039071	317.17078	0.942
M+H-H2O	6-{4-[3-(3,7-dimethylocta-2,6-dien-1-yl)-7-hydroxy-4-oxo-4H-chromen-2-yl]-3-hydroxyphenoxy}-3,4,5-trihydroxyoxane-2-carboxylic acid	1.774354	0.508532	0.01258	565.20667	5.48
M+H-H2O, M+H	Phytolaccoside A	2.392436	0.489947	0.028778	649.3934	5.595
M+H-H2O, M+H	Phenprocoumon	1.193492	0.487288	0.011956	263.10659	0.997
M+H	L-Phosphatidic acid	1.924668	0.526288	0.016282	597.37837	7.26
M+Na	Majoroside F1	1.112695	0.322465	0.015575	985.53189	8.318
M+H	11,12-Dimethoxydihydrokawain	1.2155	3.491148	0.043553	293.13775	5.205
M+H-H2O	Bambuterol	2.430262	2.084984	0.004577	350.20707	4.038
M+Na	Tamibarotene	1.432598	0.586805	0.016837	374.17108	4.453
M+H-H2O	DG(11M3/9M5/0:0)	1.263105	0.407527	0.049894	655.49102	13.429
2M+Na	2-Hydroxyflemichapparin C	1.22341	8.468334	0.046307	675.07629	2.451
M+K	Munetone	1.358837	0.127146	0.036291	455.12691	3.756
M+H	Kaempferol 3-O-sinapoyl-sophoroside 7-O-glucoside	1.349144	0.329477	0.048615	979.26758	4.182
M+H	Halobetasol Propionate	2.122594	0.204106	0.041133	485.19197	4.8
M+H	Malyngamide T	1.091776	0.480462	0.00856	468.24909	4.161
M+K	3,7-Dihydroxy-25-methoxycucurbita-5,23-dien-19-al	2.036364	2.8544	0.038622	525.33631	5.274
2M+H	Tricosanoic acid	1.923119	0.199665	0.028068	709.70485	4.325

M+H-H2O	3-Methoxymorphinan	1.994067	1.580963	0.016601	240.17468	13.406
M+NH4	Acrimarine H	1.637484	0.392369	0.011383	531.21299	5.228
M+H	CLEBOPRIDE	2.110365	1.955452	0.028942	374.16359	7.283
M+NH4	3alpha,12alpha,24R-Trihydroxy-5beta-cholestan-26-oic acid	1.211429	0.421799	0.021472	468.36807	8.247
M+Na	PS(18:3(6Z,9Z,12Z)/20:5(5Z,8Z,11Z,14Z,17Z))	1.104372	0.162784	0.028221	826.46379	8.41
M+H	Lamotrigine	1.342283	1.96835	0.022857	256.01513	0.765

Abbreviations:

[1]VIP >1 and [4]*P*-value<0.05 are listed in the table. *P* -values were calculated according to Student's T-test (n=10).

[2]FC = fold change. If the fold change value is less than 1, it means that there is less metabolite in the Mixp group than in the Ctrl group.
[3]MZ = mass-to-charge ratio.

[4]R.T = represents retention time

Fig. S1. Microbial sparsity curves of appendix contents of Shaoxing ducks with different residual feedings.

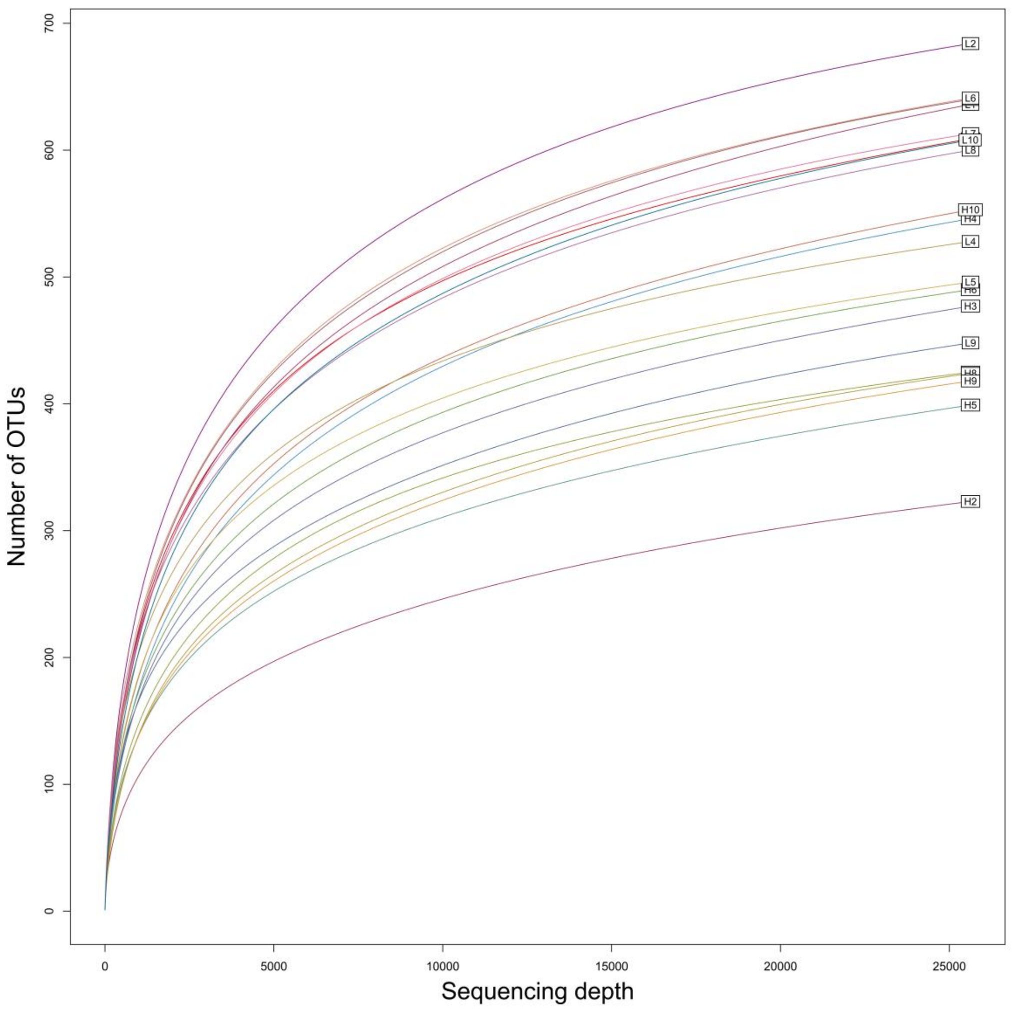
Number of OTUs
Sequencing depth
0
100
200
300
400
500
600
700
0
5000
10000
15000
20000
25000
L2
L6
L10
L8
H10
H4
L4
L5
H3
L9
H9
H5
H2
